# Connecting the conformational behavior of cyclic octadepsipeptides with their ionophoric property and membrane permeability†

**DOI:** 10.1039/d0ob01447h

**Published:** 2020-09-23

**Authors:** Thomas Stadelmann, Govindan Subramanian, Sanjay Menon, Chad E. Townsend, R. Scott Lokey, Marc-Olivier Ebert, Sereina Riniker

**Affiliations:** a Department of Chemistry and Applied Biosciences, ETH Zurich, Vladimir-Prelog-Weg 1-5, 8093 Zurich, Switzerland.; b Veterinary Medicine Research & Development, Zoetis, 333 Portage Street, Bldg. 300, Kalamazoo, Michigan 49007, USA; c Department of Chemistry and Biochemistry, University of California, Santa Cruz, California 93064, USA

## Abstract

Cyclic octadepsipeptides such as PF1022A and its synthetic derivative emodepside exhibit anthelmintic activity with the latter sold as a commercial drug treatment against gastrointestinal nematodes for animal health use. The structure-permeability relationship of these cyclic depsipeptides that could ultimately provide insights into the compound bioavailability is not yet well understood. The fully *N*-methylated amide backbone and apolar sidechain residues do not allow for the formation of intramolecular hydrogen bonds, normally observed in the membrane-permeable conformations of cyclic peptides. Hence, any understanding gained on these depsipeptides would serve as a prototype for future design strategies. In previous nuclear magnetic resonance (NMR) studies, two macrocyclic core conformers of emodepside were detected, one with all backbone amides in *trans*-configuration (hereon referred as the symmetric conformer) and the other with one amide in *cis*-configuration (hereon referred as the asymmetric conformer). In addition, these depsipeptides were also reported to be ionophores with a preference of potassium over sodium. In this study, we relate the conformational behavior of PF1022A, emodepside, and closely related analogs with their ionophoric characteristic probed using NMR and molecular dynamics (MD) simulations and finally evaluated their passive membrane permeability using PAMPA. We find that the equilibrium between the two core conformers shifts more towards the symmetric conformer upon addition of monovalent cations with selectivity for potassium over sodium. Both the NMR experiments and the theoretical Markov state models based on extensive MD simulations indicate a more rigid backbone for the asymmetric conformation, whereas the symmetric conformation shows greater flexibility. The experimental results further advocate for the symmetric conformation binding the cation. The PAMPA results suggest that the investigated depsipeptides are retained in the membrane, which may be advantageous for the likely target, a membrane-bound potassium channel.

## Introduction

Depsipeptides are atypical peptides where one or more backbone peptide amides are replaced by ester groups. Many cyclic depsipeptides were found as secondary metabolites in nature with various applications like antibiotics, antifungal and antiviral drugs, enzyme inhibitors, ionophores, anthelmintic therapeutics *etc*.^1–7^ Due to the cyclization, the flexibility of the backbone is restricted but still large enough for interaction with potential targets.^8^ This makes cyclic depsipeptides interesting lead structures for drug development.

**Scheme 1:**
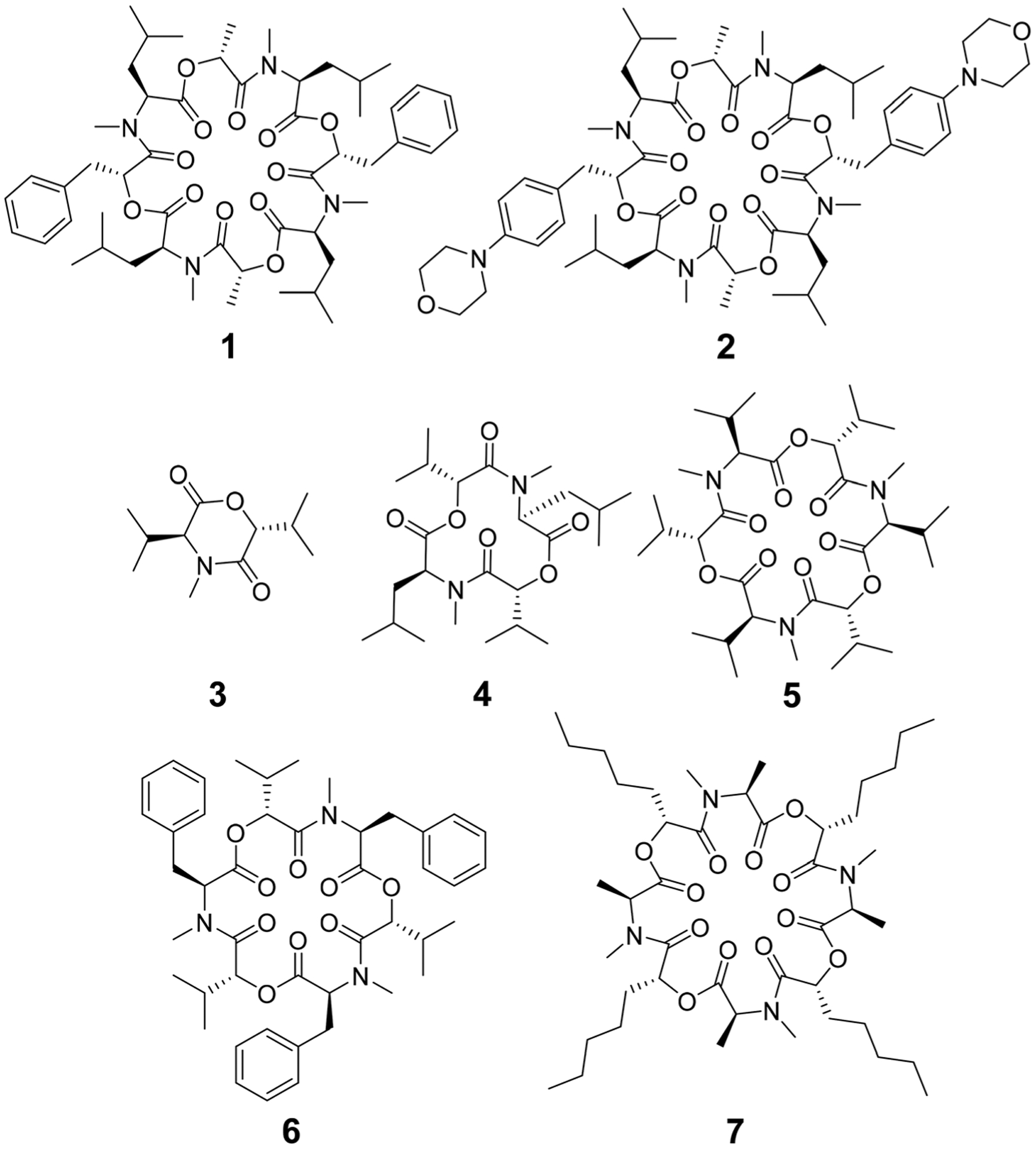
Chemical structures of cyclic depsipeptides PF1022A (**1**) consisting of four l-*N*-methyl leucines (Mle), two d-lactic acid moieties (d-Lac) and two d-phenyllactic acid moieties (d-Phl), its synthetic derivative emodepside (**2**) with two additional morpholine rings in *para* position of the phenyllactic acid residues (d-Phm), 3,6-di-(propan-2-yl)-4-methyl-morpholine-2,5-dione (**3**), cyclo-(*N*-methyl l-leucine d-hydroxyisovaleric acid)_2_ (**4**), enniatin B (**5**) consisting of three repetitions of l-*N*-methyl valine and d-hydroxyisovaleric acid, beauvericin (**6**) consisting of three repetitions of l-*N*-methyl phenylalanine and d-hydroxyisovaleric acid and verticilide (**7**) consisting of four repetitions of l-*N*-methyl alanine and d-2-hydroxyheptanoic acid.

The cyclic octadepsipeptide PF1022A (**1**) (Scheme 1) is a natural product, consisting of two repetitions of d-lactic acid, *N*-methyl l-leucine, d-phenyllactic acid and *N*-methyl l-leucine and has therefore a *C*_2_ symmetry axis. PF1022A demonstrates pharmacological activity against nematodes.^9^ Its synthetic derivative, emodepside (**2**) (Scheme 1), containing additional morpholine rings at the *para* position of the phenyllactic acid aromatic rings, exhibits increased anthelmintic activity^10^ and is a commercial drug effective against a number of gastrointestinal nematodes in cats. PF1022A and emodepside belong to a subfamily of cyclic depsideptides that have all the backbone amides methylated and possess only apolar side chains. This means that no hydrogen bond donors are present and thus, the formation of intramolecular hydrogen bonds is not possible. Yet, the ability to adopt a conformation, which maximizes the number of intramolecular hydrogen bonds is thought to be essential for passive membrane permeation of cyclic peptides.^11–17^ Nevertheless, some members of this subfamily of cyclic depsipeptides were found to be permeable or can be easily incorporated into a lipid membrane.^18^ Thus, to exploit their potential as therapeutics, it is important to establish a better understanding of the relationship between structure (conformational behavior) and permeability.

Some of the known members of the subfamily of fully backbone *N*-methylated cyclic depsipeptides with varying core ring sizes are shown in Scheme 1. The smallest members consist only of one *N*-methylated amino acid and one hydroxy acid (*n* = 1). For example, 3,6-di-(propan-2-yl)-4-methyl-morpholine-2,5-dione (**3**) is a natural product and was identified as a potential precursor of the cyclic hexadepsipeptide enniatin B (**5**).^19,20^ It showed moderate antioxidant and antimicrobial activity.^21,22^ Structurally, both the amide and the ester bond in the six-membered ring are in *cis*-configuration.^20^ The next larger members consist of two repetitions of an amino acid and a hydroxy acid (*n* = 2). In NMR studies of cyclo-(*N*-methyl l-leucine hydroxyisovaleric acid)_2_ (**4**) in chloroform, a *C*_2_-symmetric conformation was observed.^23,24^ In contrast to enniatin B (**5**) (*n* = 3), it showed no activity against mycobacteria.^24^ Enniatin B consists of three *N*-methyl l-valine and three d-hydroxyisovaleric acids and adopts, based on NMR studies, a *C*_3_-symmetric conformation with all amides in *trans*-configuration in chloroform.^25^ It is a well-known antibacterial, anthelmintic, antifungal, herbicidal and insecticidal compound.^26^ Due to the lipophilic nature of **5**, it can be easily incorporated into lipid bilayers of cell membranes. Enniatin B was found to be ionophoric, *i.e.* it can carry mono and divalent cations through membranes with a selectivity for K^+^ over Na^+^.^25,26^ Further, it can form stable complexes with cations in solution. A 1 : 1 as well as a 2 : 1 sandwich (peptide : cation ratio) complex were observed.^27^ A 3 : 2 complex was proposed as well but with lower stability than the 1 : 1 and the 2 : 1 complexes.^27^ Enniatin B showed decent permeability (log *P*_e_ = −4.73) in a passive artificial membrane permeability assay (PAMPA)^28^ and a permeability of 6.1 × 10^−4^ cm s^−1^ in a Caco-2 permeability assay.^18,29^ Beauvericin (**6**) belongs, like **5**, to the enniatin family. It consists of three alternating *N*-methyl l-phenylalanine and d-hydroxyisovaleric acid residues and was observed in NMR experiments to adopt a *C*_3_-symmetric conformation with all amides in *trans*-configuration in chloroform.^1,30^ **6** shows cytotoxic, apoptotic, anticancer, anti-inflammatory, antimicrobial, insecticidal and nematocidal activities and is able to transport cations, particularly Ca^2+^ through lipid bilayers.^31^ The passive membrane permeability of **6** was determined to be 5.8 × 10^−4^ cm s^−1^ in a Caco-2 permeability assay,^29^ which is similar to the permeability of **5**. Verticilide (**7**) is a cyclic octadepsipeptide (*n* = 4) such as PF1022A (**1**) and emodepside (**2**), and consists of four repetitions of *N*-methyl l-alanine and four repetitions of d-2-hydroxyheptanoic acid.^32^ **7** was found to be a ryanodine-binding inhibitor and appears in NMR experiments in chloroform as two - not further studied - conformations in a ratio of 3 : 4.^32^ Simplification of the NMR spectra of **7** was observed after the addition of a 100-fold excess of KSCN and only one conformer was detected.^32^

**Scheme 2:**
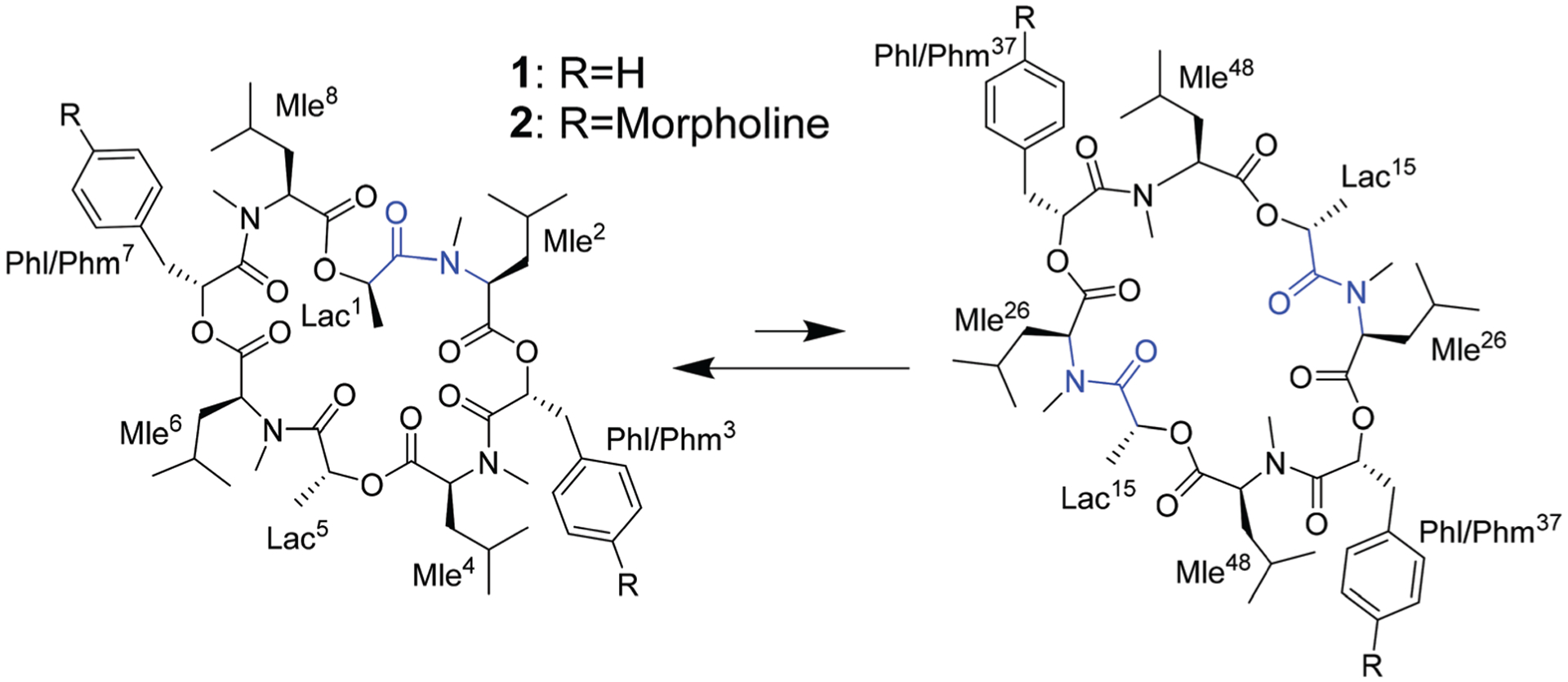
Asymmetric (left) and symmetric (right) conformations of the two cyclic octadepsipeptides PF1022A (**1**) and emodepside (**2**) consisting of four l-*N*-methyl leucines (Mle), two d-lactic acid moieties (d-Lac) and two d-phenyllactic acid moieties (d-Phl) (with additional morpholine rings in *para* position in case of **2** (d-Phm)). In the *C*_2_ symmetric conformation, the chemically equivalent residues share a common designation derived from their position in the asymmetric conformation.

The investigated compounds are only poorly soluble in water. Therefore, methanol and chloroform were chosen as simple mimics for a polar environment and the cell membrane, respectively. In both solvents, the NMR spectra of PF1022A (**1**) revealed two main conformations that interconvert slowly on the NMR time-scale (Scheme 2). The conformer ratio of **2** has been reported to be 4 : 1 in methanol and 3 : 1 in chloroform in previous studies.^9,33^ The major conformation is characterized by a single *cis*-amide bond between the d-lactic acid and the *N*-methyl-l-leucine residue and is thus named *asymmetric*, whereas all amide bonds are *trans* in the minor conformation, thus named *symmetric*.^9,33^ The crystal structure of **1** apparently shows the asymmetric conformation, however, the data was not deposited with the CCDC (CCDC code MORJEI).^34^ The crystal structure of **2**, on the other hand, is the symmetric conformation (CCDC code DOMZOW).^35^

**Scheme 3:**
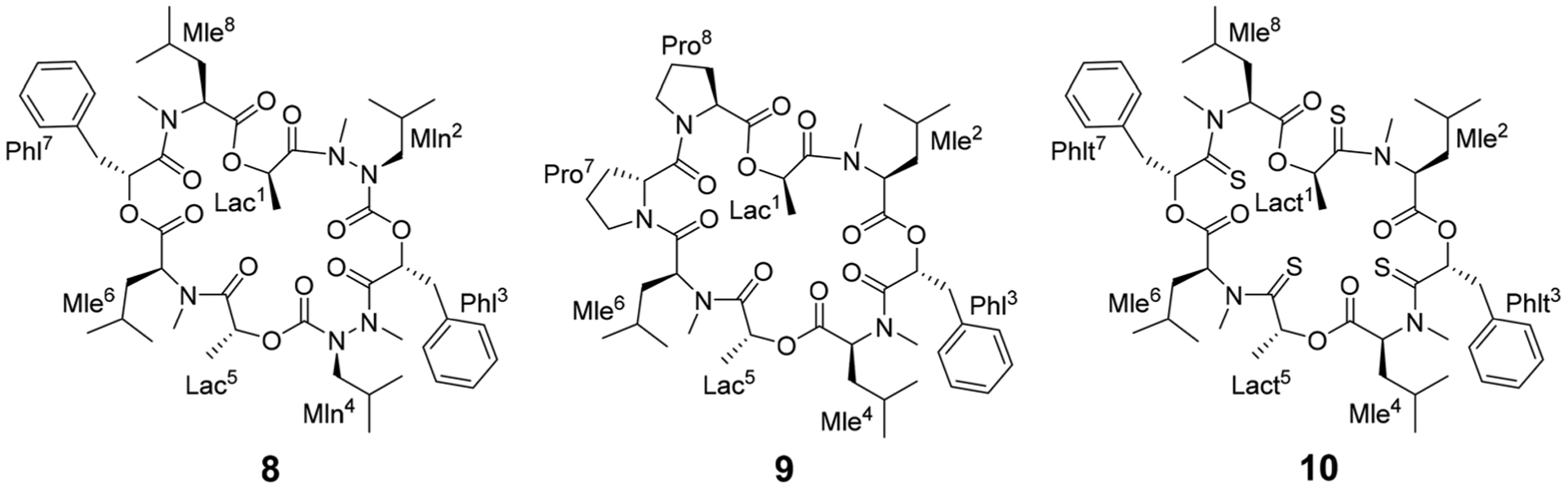
Chemical structures of the bis-aza PF1022A analog (**8**) in which two Cα carbons in *N*-methyl residues are replaced by nitrogens (Mln), of the di-proline PF1022A analog (**9**), in which residues 7 and 8 are replaced by a turn inducing d-Pro l-Pro moiety and of a tetra thioamide PF1022A analog (**10**) in which lactate and phenyllactic acid residues are replaced by their corresponding thio-analogs (Lact and Phlt).

Different side-chain and backbone modifications of **1** have been investigated in the literature.^33,36–38^ An interesting compound with regard to its conformational behavior is the bis-aza analog of PF1022A (**8**) (Scheme 3), where the asymmetric conformation is stabilized with a 100 : 7 conformer ratio in chloroform.^36^ The conformation solved in the crystal structure is also asymmetric (CCDC code QOXDOW).^36^ The biological activity of **8** was found to be weaker by a factor of 5–10 compared to **1**.^36^ For another modification with a turn-inducing element consisting of two prolines (d-Pro-l-Pro) (**9**) (Scheme 3), it was reported that the symmetric conformation is stabilized such that only this conformer is present in solution.^37^ Furthermore, the biological activity of **9** was found to be higher by a factor of 2 compared to **1**.^37^ These observations led to the hypothesis that the propensity for the symmetric conformation is crucial for anthelmintic activity. However, for a third modification of **1**, in which the four peptide bonds were replaced by thiopeptide bonds (**10**) (Scheme 3), the activity was also increased 2.5 times compared to **1**.^38^ In this case, the increased activity was attributed to a more rigid asymmetric conformation due to the *N*-methyl-thioamides, which stabilize the *cis*-amide bond between d-thiolactic acid and *N*-methyl-l-leucine.^33,38^ Based on the published data, no clear correlation between activity and conformational preference for the symmetric or asymmetric structure can be found, especially if it is considered that an increase or decrease of activity by a factor of 2 is mostly within the accuracy of experiment. Additionally, no experimental membrane permeability data for **1**, **2** and **8–10** is reported in the literature.

The mechanism of action of **1**, **2** and related compounds is not yet fully understood. Initially, their anthelmintic activity was attributed to the binding of a presynaptic latrophilin receptor.^10^ More recently, binding to the calcium-activated potassium channel SLO-1 was proposed to be involved in the activity of **2**, possibly in combination with the latrophilin receptor.^39–42^ No crystal structure of **1** or **2** bound to one of these proteins is available. PF1022A and derivatives were reported to be ionophores with selectivity for K^+^ over Na^+^,^43^ similar to enniatin B. However, the ion carrier property across lipid bilayers does not appear to be related to the anthelmintic activity, because the enantiomer of PF1022A (*i.e.* all d- and l-residues switched) exhibited the same ionophoric ability but no anthelmintic activity.^43^

In this study, the interplay between the macrocyclic core conformational behavior of PF1022A, emodepside and related compounds with their ionophoric nature and their passive membrane permeability was investigated to enhance our understanding for the rational design of such cyclic octadepsipeptides with improved profiles. For this, we characterized the conformational behavior of **1**, **2** and **8** and the effect of monovalent cations on the conformational ensembles using solution NMR measurements and extensive MD simulations in chloroform and methanol. With this data, we want to explore how the cyclic depsipeptides interact with cations and determine a plausible coordination mode. The complexation with a cation could be an effective mechanism to bury the polar groups and thus, may be a crucial step for the incorporation of the depsipeptides into the membrane. The passive membrane permeability is assessed with PAMPA with and without the addition of potassium.

## Results and discussion

### Characterization of the conformational behavior

#### NMR measurements in methanol and chloroform.

NMR spectra of **1**, **2** and **8** were recorded in CD_3_OH and CDCl_3_. The conformer ratios (Table 1) are in good agreement with those reported previously in the literature.^9,33,36^ A small batch-to-batch variability in the conformer ratio of **1** (ratios between 5 : 1 to 7 : 1 in methanol) was observed. The assignment of the major and minor conformer of **1** and **2** as well as of the major conformer of **8** in CDCl_3_ and CD_3_OH including proton, carbon and partly also nitrogen chemical shifts can be found in the ESI.†

**Fig. 1:**
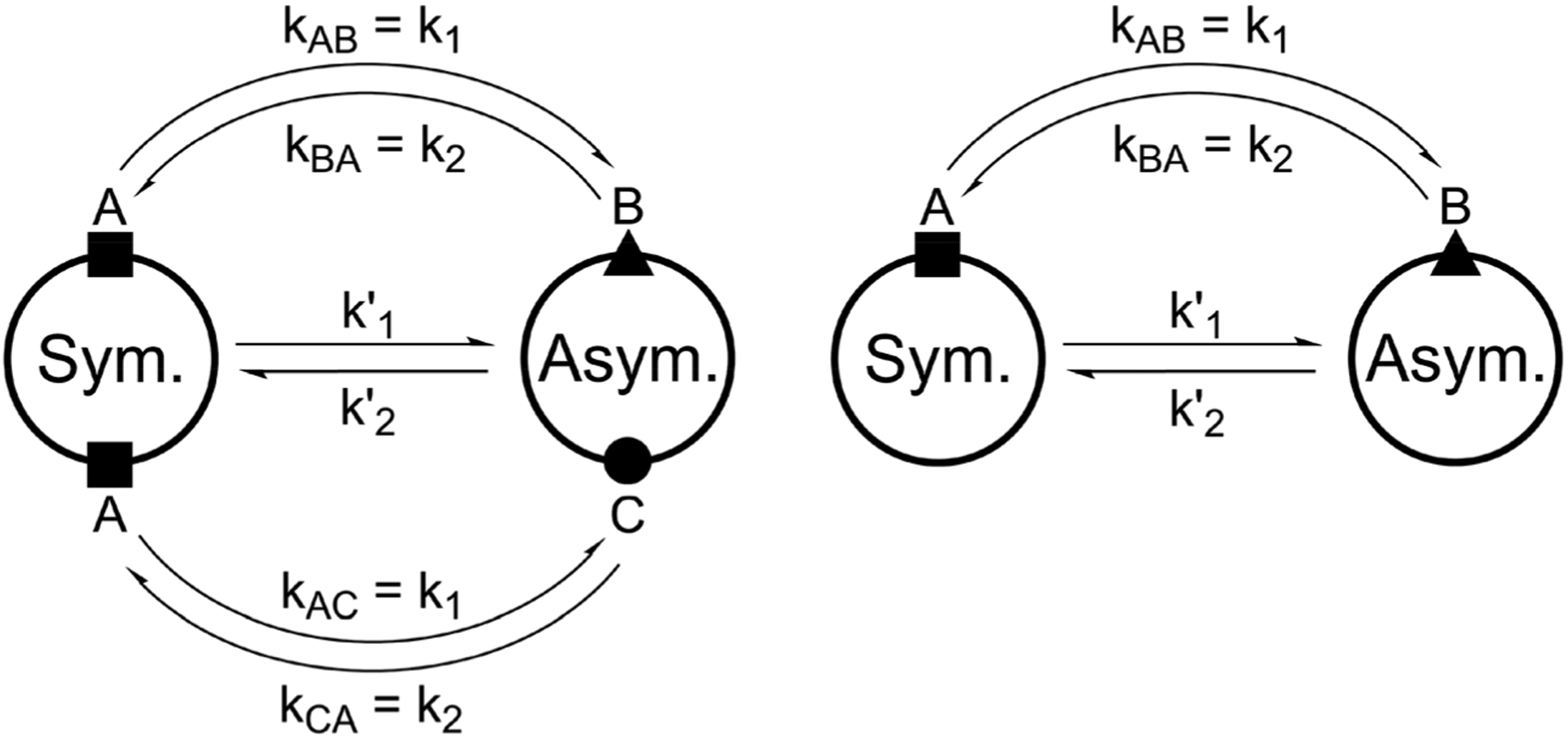
Schematic drawing of the magnetization transfer pathways used for the analysis of the EXSY data for **1**, **2** (left) and **8** (right). In the symmetric conformation of **1** and **2**, one of the two chemically equivalent amide bonds can flip into a *cis*-configuration to reach the asymmetric conformation (amide bond between Lac^15^ and Mle^26^, see Scheme 1). In this process, magnetization is transferred *via* two different site-to-site pathways (A ↔ B and A ↔ C with *k*_AB_ = *k*_AC_ = *k*_1_ and *k*_BA_ = *k*_CA_ = *k*_2_), each leading to a separate set of EXSY cross-peaks. During a transition from the symmetric to the asymmetric conformation, each nucleus in a symmetric pair undergoes either pathway equally likely. In the reverse process from the asymmetric to the symmetric conformation a nucleus at site B will always follow A ↔ B whereas a nucleus at site C will always follow A ↔ C. As a consequence, the site-to-site exchange rates *k*_1_ and *k*_2_ for **1** and **2** differ from the mechanistic exchange rates: *k*’_1_ = 2 × *k*_1_ and *k*’_2_ = *k*_2_ where *K* = *k*’_1_/*k*’_2_. In **8**, the *C*_2_ symmetry is broken by the two additional nitrogen atoms in the backbone and only a single magnetization transfer pathway has to be considered. Therefore for **8** *k*_1_ = *k’*_1_ and *k*_2_ = *k’*_2_.

For all the investigated peptides, exchange peaks (EXSY peaks) could be detected in ROESY spectra recorded in chloroform-d. In CD_3_OH, EXSY peaks could only be detected for **8** but low intensity and limited resolution did not allow further analysis. Besides the expected EXSY cross-peaks between the major asymmetric and the minor symmetric conformer for **1** and **2**, additional EXSY peaks are present, which indicate that more than the two known conformations are populated in solution. At least two additional low-intensity conformers could be identified (see ESI†). Using the volumes of the EXSY cross-peaks it is possible to calculate the site-to-site exchange rates *k*_1_ and *k*_2_ between the magnetic sites in the interconverting conformers (Fig. 1). The additional two low-intensity conformers were neglected in the calculation of the exchange rates since their intensity was close to the noise level and their corresponding diagonal peaks were partially buried under other, more intense signals. The calculated site-to-site rates are summarized in Table 2.

The site-to-site exchange rates of **1**, **2** and **8** are comparable and are about twice as high compared to the exchange rate reported for cyclosporine A (*k*_ex_ ≈ 0.1 s^−1^).^44^ This is plausible as the smaller ring size of the cyclic octadepsipeptides (24-membered ring) compared to cyclosporine A (33-membered ring) increases the ring strain. Since these results are based on a single mixing time, no direct error estimate can be given. From the comparison of the cross-peak intensities on both sides of the diagonal, errors about 20% can be assumed.

**Fig. 2:**
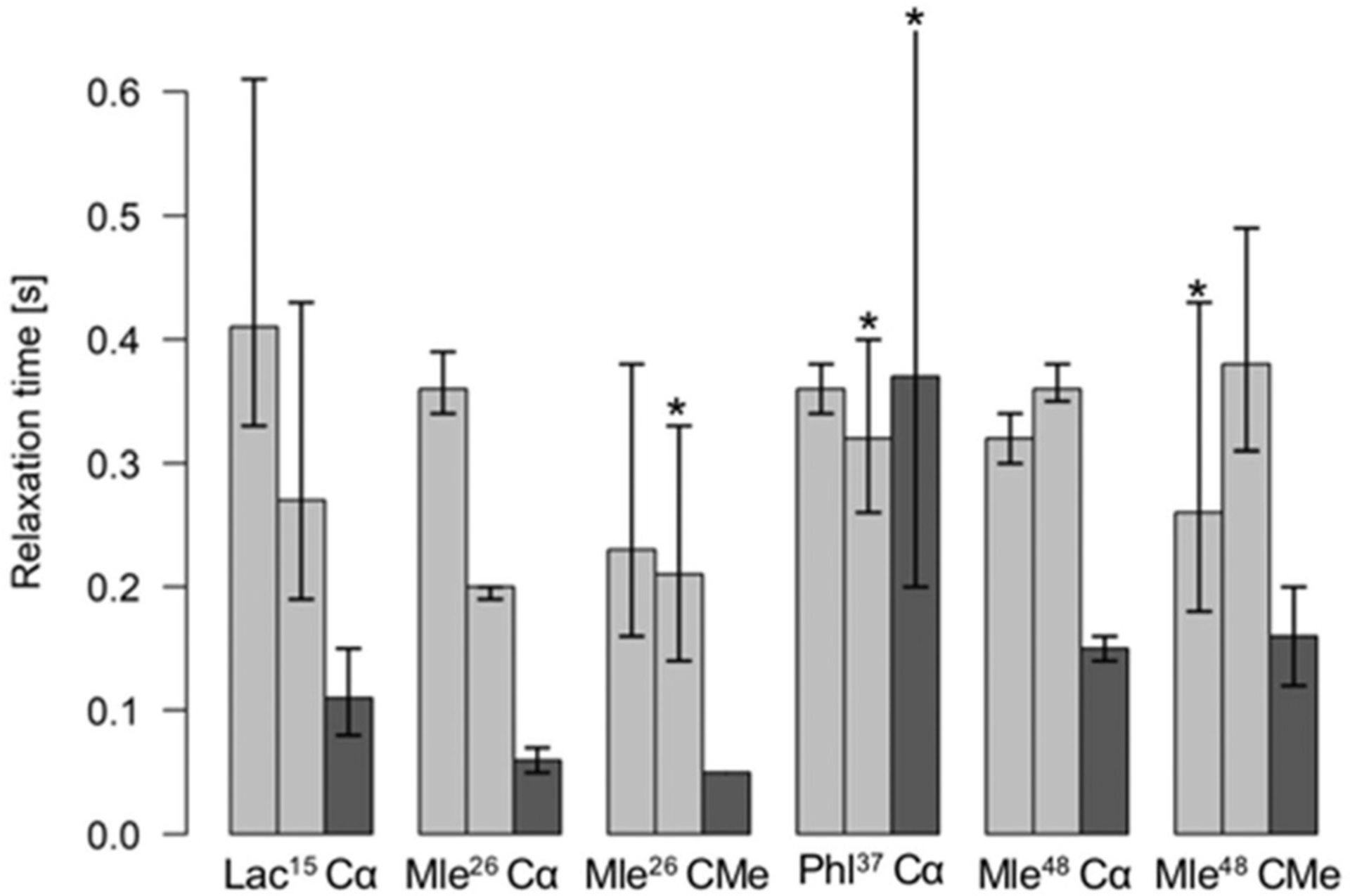
^13^C *T*_2_ relaxation times measured for 20 mM PF1022A (**1**) in CDCl_3_ with a series of ^13^C-CPMG HSQC spectra with relaxation delays from 15.2 to 456 ms and with compensation of heating effects. Entries marked with * belong to partly overlapping peaks. The first two light grey bars belong to the asymmetric conformation (*i.e.* Lac^1^ Cα and Lac^5^ Cα) whereas the third bar (dark grey) belongs to the symmetric conformation (*i.e.* Lac^15^ Cα). Error bars indicate the 95% confidence interval of the fit.

In ^1^H and ^13^C NMR spectra of the investigated cyclic octadepsipeptides, the signals for the symmetric conformer were generally found to be broader. To quantify this additional exchange broadening, presumably originating from processes on the millisecond to microsecond range, ^13^C *T*_2_ relaxation time measurements of **1** in CDCl_3_ were performed (Fig. 2). It is clearly visible that the symmetric conformer has shorter *T*_2_ relaxation times for the backbone carbons compared to the asymmetric conformer. This indicates greater backbone flexibility on the μs to ms timescale for the symmetric conformer. To the best of our knowledge, this is the first time that such behavior was observed for a cyclic depsipeptide.

#### Kinetic models based on molecular dynamics (MD) simulations.

Extensive MD simulations of **1**, **2** and **8** were performed in methanol and chloroform using the GROMOS simulation package^45^ and the GROMOS 54A7 united-atom force field.^46^ As starting structures, the symmetric crystal structure of emodepside (**2**) (CCDC code DOMZOW) and the asymmetric crystal structure of the bis-aza analog (**8**) (CCDC code QOXDOW) were used. No significant differences in the structural ensemble could be detected between them. In general, the symmetric backbone configuration was found to be over stabilized in the MD simulations, although the asymmetric configuration is more stable according to NMR. No *trans*-to-*cis* isomerizations were observed in the simulations, whereas five *cis*-to-*trans* isomerizations occurred. This is likely a force field issue, because *trans*-amide bonds are generally preferred in protein crystal structures. As a proof-of-principle, the partial charges in the methylated amide were slightly redistributed, which reduced the *cis*-to-*trans* isomerization rate substantially. As isomerizations are generally a rare event in finite simulations, we decided to analyze the symmetric and asymmetric conformations separately while assuming that the conformer distributions within the two sub-ensembles are correctly reproduced in the MD simulations.

**Fig. 3:**
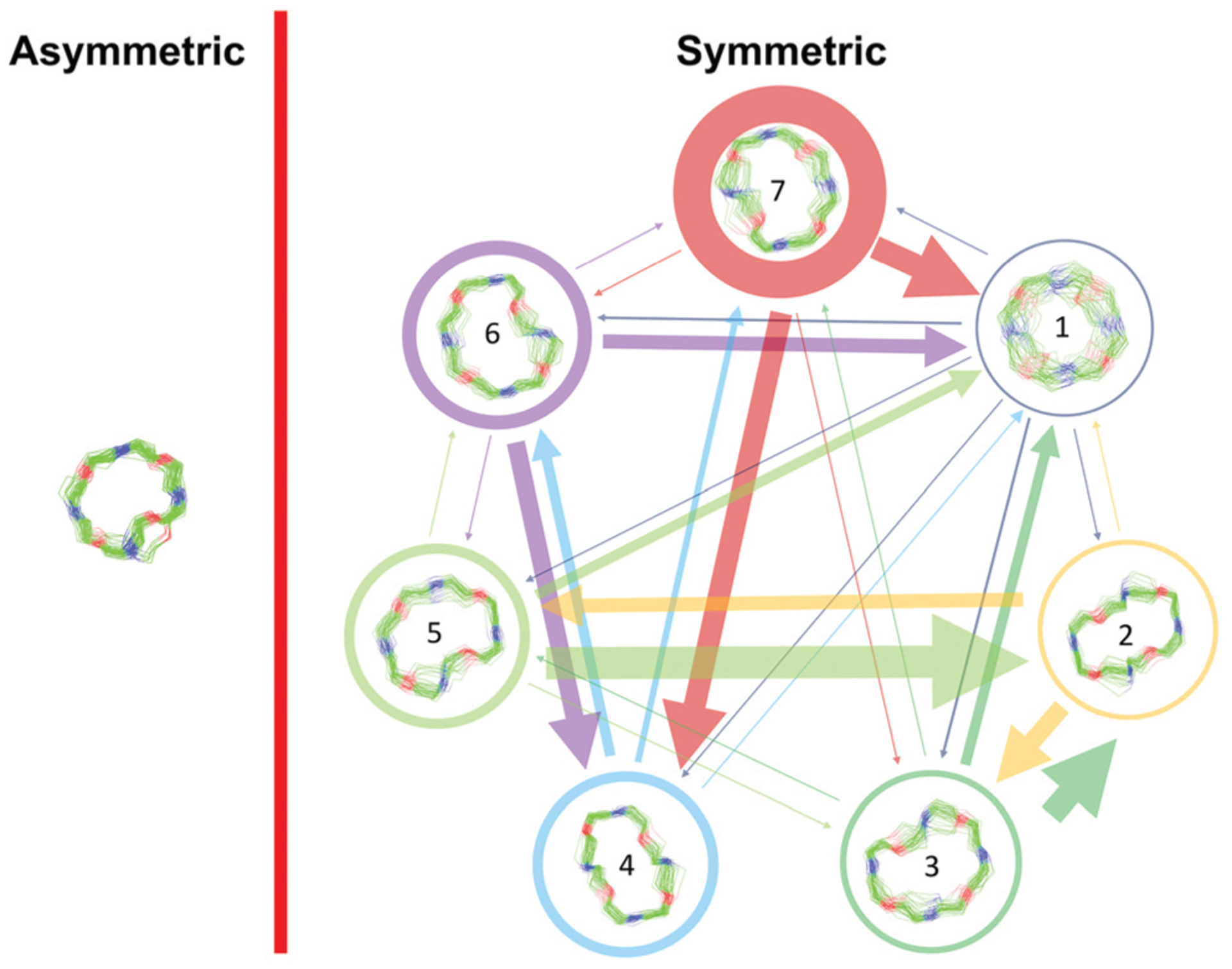
Visualization of the MSMs of the asymmetric and symmetric subsets of PF1022A (**1**) in chloroform. For each conformational state, 50 randomly picked backbone structures are shown. The thickness of the circle surrounding the state indicates the corresponding population with state 1 as the least and state 7 as the most populated conformational state. Note that state 3 and 5 as well as state 6 and 7 are chemically the same due to the *C*_2_ symmetry of the symmetric conformation. The equilibrium populations are 7.4% and 11.9% for states 3 and 5, respectively, and 16.5% and 43.2% for states 6 and 7, respectively. A likely issue is that all simulations were started from the two available crystal structures. The arrows indicate the transition probabilities for state i going to state j within the chosen lag time (*i.e.* 10 ns). The arrow size corresponds to the magnitude of the probability. The subsets were analyzed separately because not enough transitions between symmetric and asymmetric conformers were observed.

Markov state models (MSMs)^47–50^ are a powerful tool to analyze the conformational dynamics in MD simulations. Here, we generated core-set Markov models of PF1022A (**1**) in chloroform using common nearest neighbor (CNN) based clustering^49,51–53^ and the PyEMMA package.^54^ This procedure has been used successfully with other cyclic peptides.^12^ The MSMs were constructed separately for the asymmetric and the symmetric conformations (but with the same TICA space^55^). For the asymmetric subset, only two unconnected conformational states could be identified, whereby one arose from a single simulation and was considered as noise. Therefore, the backbone with the asymmetric configuration appears to be relatively rigid. In contrast, the backbone with the symmetric configuration shows substantially more flexibility, and seven conformational states could be observed (Fig. 3). This is in line with the NMR experiments, where shorter *T*_2_ relaxation times were observed for the symmetric conformer, indicating higher flexibility on the μs-ms time scale.

The conformational states 3 and 5, as well as 6 and 7, are in principle the same, rotated by 180° due to the *C*_2_ symmetry of the symmetric conformation. This allows for an easy check of convergence. It can be seen in Fig. 3 (and Table S8 in the ESI†) that the model is not yet fully converged. Note that the starting structure of the simulation corresponds to state 7. Conversion from state 7 to state 6 is essentially a complete reorientation of the entire backbone. Thus, very long simulations (>10 μs) would be needed to obtain the same population for state 6. Nevertheless, the results also indicate that the conformational space for the symmetric conformation is already sampled quite extensively.

### Effect of the presence of monovalent cations

#### Binding affinity and conformer ratio as a function of the cation concentration.

**Fig. 4:**
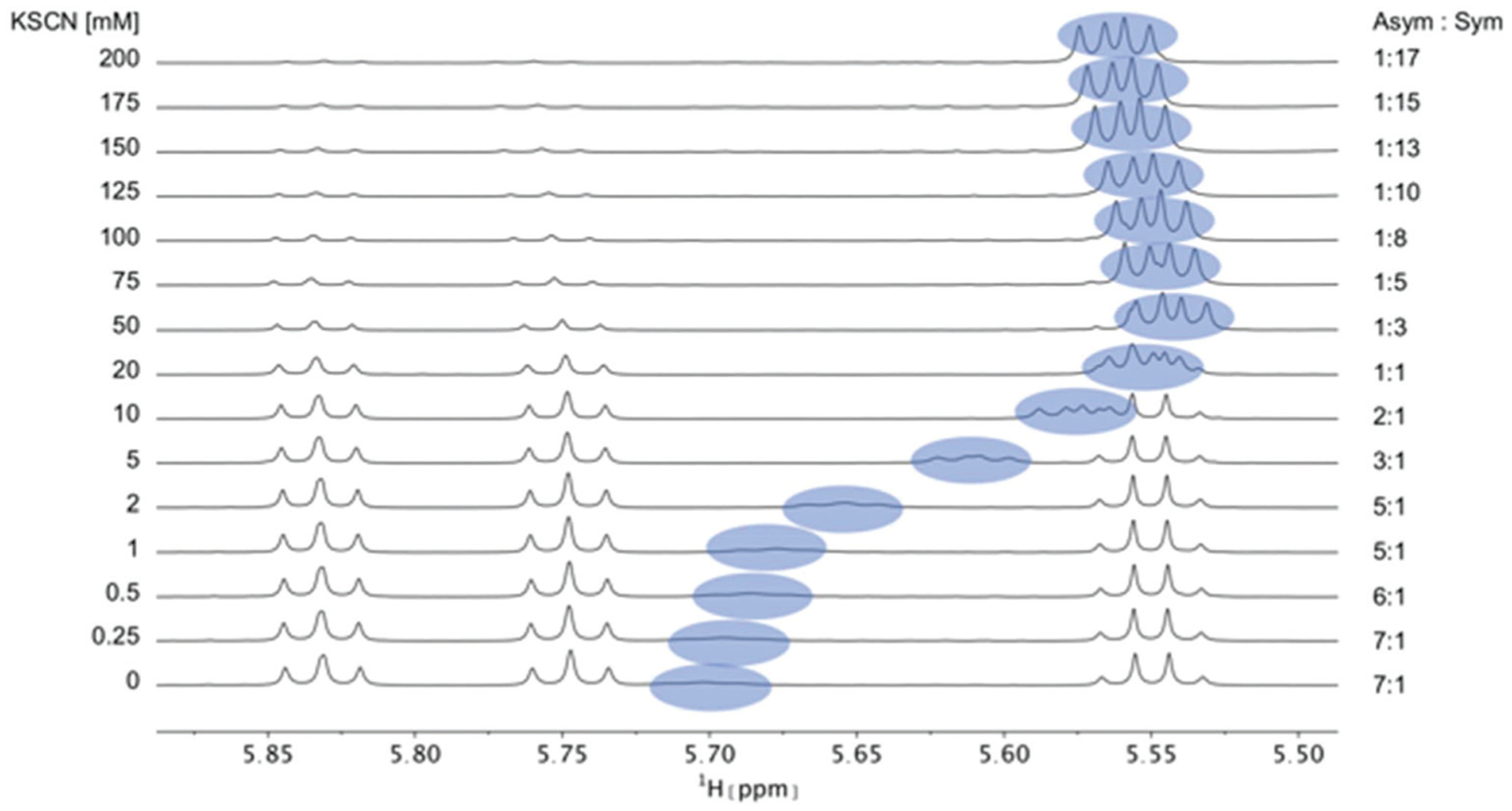
Titration of 5 mM PF1022A (**1**) with a KSCN solution in CD_3_OH: Hα region of ^1^H NMR spectra. Chemical shift changes were observed for the symmetric conformation, best seen for the signal of the Hα proton in residue Phl^37^ (blue labels). In addition, a change in the ratio between the symmetric and asymmetric conformation is observed. Also the asymmetric conformation shows small changes in chemical shift at high salt concentrations. The titration plot for emodepside (**2**) can be found in the ESI.†

PF1022A (**1**) was previously reported to bind monovalent cations and act as an ionophore. However, no direct relationship between the ionophoric property and anthelmintic activity was found.^43^ Further, simplification of NMR spectra was observed upon addition of KSCN but never described in any detail.^32^ On the other hand, the connection of ion binding with the macrocycle conformational behavior as well as its importance for the membrane permeability is not yet clear. Therefore, we recorded NMR spectra in methanol of **1** and **2** in the presence of different concentrations of KSCN and NaSCN (in the case of **1** also NH_4_SCN and CsSCN). In addition, the bis-aza analog **8** was titrated with KSCN. A significant change in chemical shift for the symmetric conformation was observed for **1** and **2** upon addition of the salts, with the effect being most pronounced for Cs^+^ followed by K^+^, Na^+^ and NH ^+^_4_. The cation preference is in line with a previous study.^43^ The change in chemical shift can be seen best for the Hα proton of Phl^37^ in **1** (Fig. 4), and the Hα proton of Phm^37^ in **2** (Fig. S7 in ESI†). In addition, the ratio between the asymmetric and the symmetric conformer changes dramatically in favor of the symmetric conformation with increasing cation concentration (Table 3). Such a restriction to a single conformer was also seen for verticilide (**7**) upon addition of KSCN,^32^ although no structural characterization was done in that case.

**Fig. 5:**
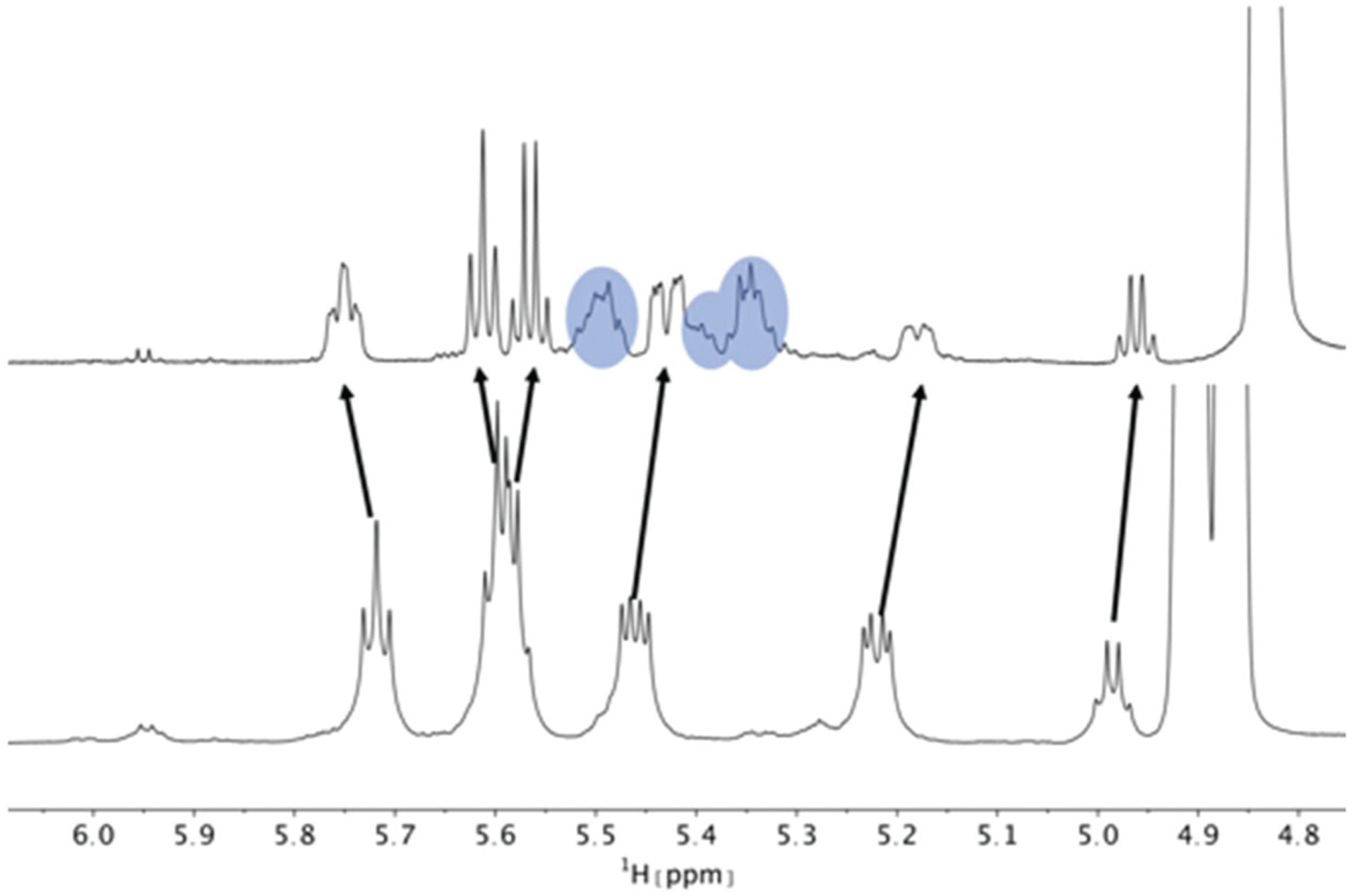
Hα region of the ^1^H NMR spectra of a 5 mM solution of the bis-aza analog (**8**) without (bottom) and with 200 mM KSCN (top) in CD_3_OH. Chemical shift changes were observed for the asymmetric conformation. Compared to **1** and **2**, the change in ratio between asymmetric and symmetric conformation is less pronounced and is close to 1 : 1 at a 40-fold excess of KSCN. Peaks of the symmetric conformation are marked in blue. The arrows indicate the movement of the asymmetric peaks upon addition of KSCN. On the right, the residual solvent peak is visible.

The changes in asymmetric : symmetric ratio upon addition of monovalent salt are comparable between **1** and **2** for KSCN and NaSCN. Consequently, the affinities of the two peptides for the cations are expected to be very similar. Therefore, for subsequent titrations only PF1022A (**1**) was used. In contrast, the bis-aza analog (**8**), which predominantly adopts the asymmetric conformer, required a much higher salt concentration to observe a shift in the conformer ratio (Fig. 5).

**Fig. 6:**
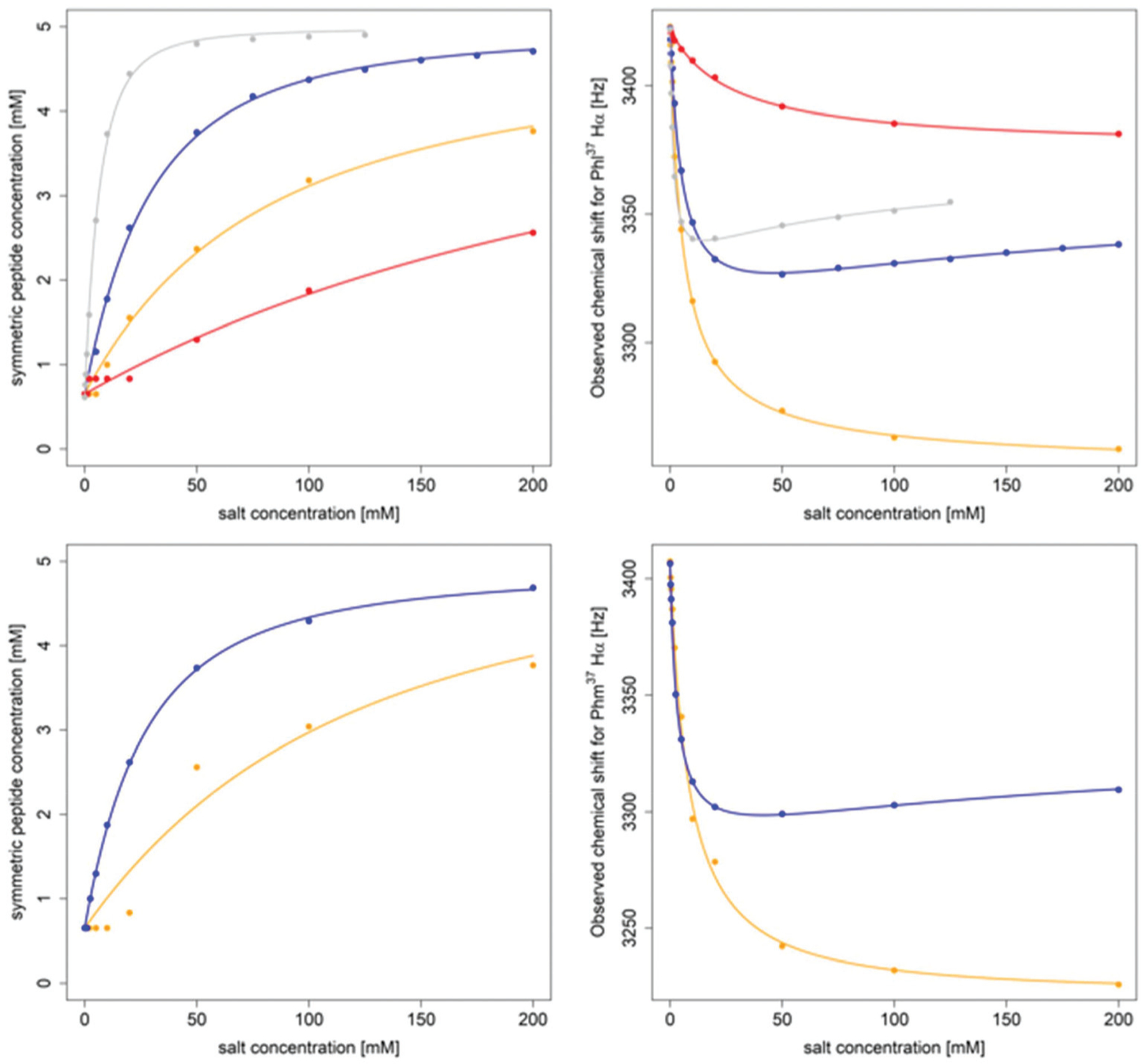
Titration of 5 mM PF1022A (**1**) (top) and 5 mM emodepside (**2**) (bottom) with different monovalent cations (CsSCN in grey, KSCN in blue, NaSCN in orange and NH_4_SCN in red) in CD_3_OH while the total volume was kept constant. The titration with CsSCN was only done up to 125 mM due to solubility issues. (Left): Change of the concentration of the symmetric conformation upon the addition of the corresponding salt. The data points were fitted with a damped logistic growth function (for details see ESI†). (Right): Change of the chemical shift of the Phl^37^/Phm^37^ Hα proton as a function of the salt concentration (for details of the fit, see ESI†). The plots were generated with *R*.^58^

The titration data of PF1022A (**1**) with KSCN and CsSCN (as well as **2** with KSCN) can only be explained by a model containing at least two different ion-bound symmetric species, which are in fast exchange with the unbound symmetric conformation. In the case of a simple mixture of the free depsipeptide and only a 1 : 1 complex, the observed chemical shift is expected to change from the value of the free conformer towards that of the ion-bound conformer. However, we do not observe this asymptotic behavior. Instead, first the chemical shift drops with increasing salt concentration, then reaches a minimum and increases again at high concentrations. This indicates that at least a third symmetric species, which interacts with the ion, is populated. We propose a mixture of a 2 : 1 (peptide : cation ratio) and a 1 : 1 complex in solution, as was reported for enniatin B (**5**) and beauvericin (**6**).^25,27^ Such a mixture was already postulated for PF1022A (**1**) but not supported by any experimental data.^56^ Normally, fitting of the equilibrium constants *K*_1_ and *K*_2_ is straightforward using the measured change in chemical shift in dependence of the salt concentration.^57^ However, this system is more complicated due to the pre-equilibrium between the free asymmetric and symmetric conformers, and possibly additional species such as a 2 : 1 complex with one symmetric and one asymmetric conformer, or an asymmetric ion-bound conformer. We fitted our data with a model containing the free peptide in its symmetric conformation, the symmetric 1 : 1 complex, and the symmetric 2 : 1 complex. Instead of explicitly considering the pre-equilibrium, we have used the total concentration of all symmetric species obtained from integration of the ^1^H spectra. We interpret the results only qualitatively since similar fits may be achieved with different fitting parameters. Fig. 6 clearly shows that the change in the asymmetric : symmetric ratio can be used to qualitatively measure the cation affinity of the symmetric conformer. The order of affinities with Cs^+^ > K^+^ > Na^+^ > NH_4_^+^ is in agreement with those reported in literature,^43^ where alkali metals from Li^+^ to Cs^+^ were tested. If the change in chemical shift is plotted as a function of the salt concentration while keeping the peptide concentration constant, it can be observed that the change in chemical shift at high salt concentration is ordered by cation size. One could therefore speculate that the backbone of the depsipeptide has to adapt more extensively to accommodate smaller ions. This, in turn, leads to larger chemical shift changes in these complexes.

A consistent pattern is visible when comparing the plots on the left side and on the right side of Fig. 6. A higher salt concentration is needed to achieve a 1 : 1 ratio between the asymmetric and the total symmetric species than for a 50% change in chemical shift. The apparent lag increases with decreasing ion affinity. One can show that this behavior can already be reproduced by two coupled equilibria (ion independent conformational change and formation of the 1 : 1 complex). Its observation alone does not imply any cooperative phenomena or the presence of higher order complexes. Without further knowledge about the relative stabilities of the 2 : 1 and 1 : 1 complexes for each metal, a more detailed analysis is not possible at this stage.

**Fig. 7:**
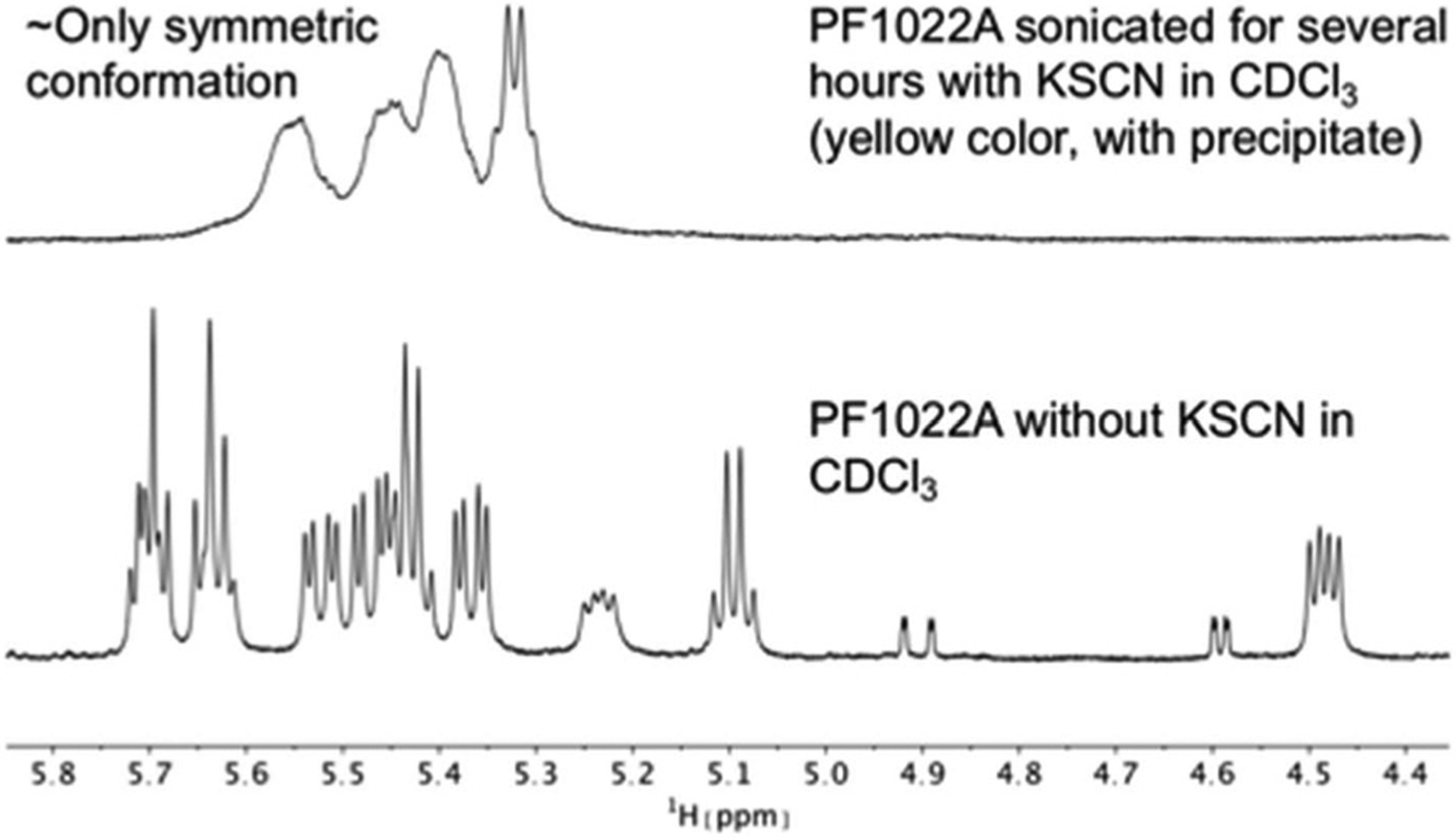
Comparison of ^1^H NMR spectra of the Hα region of PF1022A (**1**) in CDCl_3_ measured on a 500 MHz spectrometer. After the addition of KSCN and sonication, the symmetric conformation is present almost exclusively in solution. Note that the solution with the precipitate turned yellow.

It is known that valinomycin, a cyclic dodecadepsipeptide, as well as some crown ethers can bind cations even in an apolar solvent.^59–61^ This ability is an indirect evidence that the ion-bound complex may exist inside the membrane interior, *i.e.* that ion transport across a membrane is possible. To assess if the cyclic octadepsipeptides are also able to bind cations in an apolar solvent, KSCN was added to a solution of **1** in chloroform and sonicated for several hours. In subsequent NMR measurements, only the symmetric conformation could be detected in solution (Fig. 7), which indicates ion binding.

**Fig. 8:**
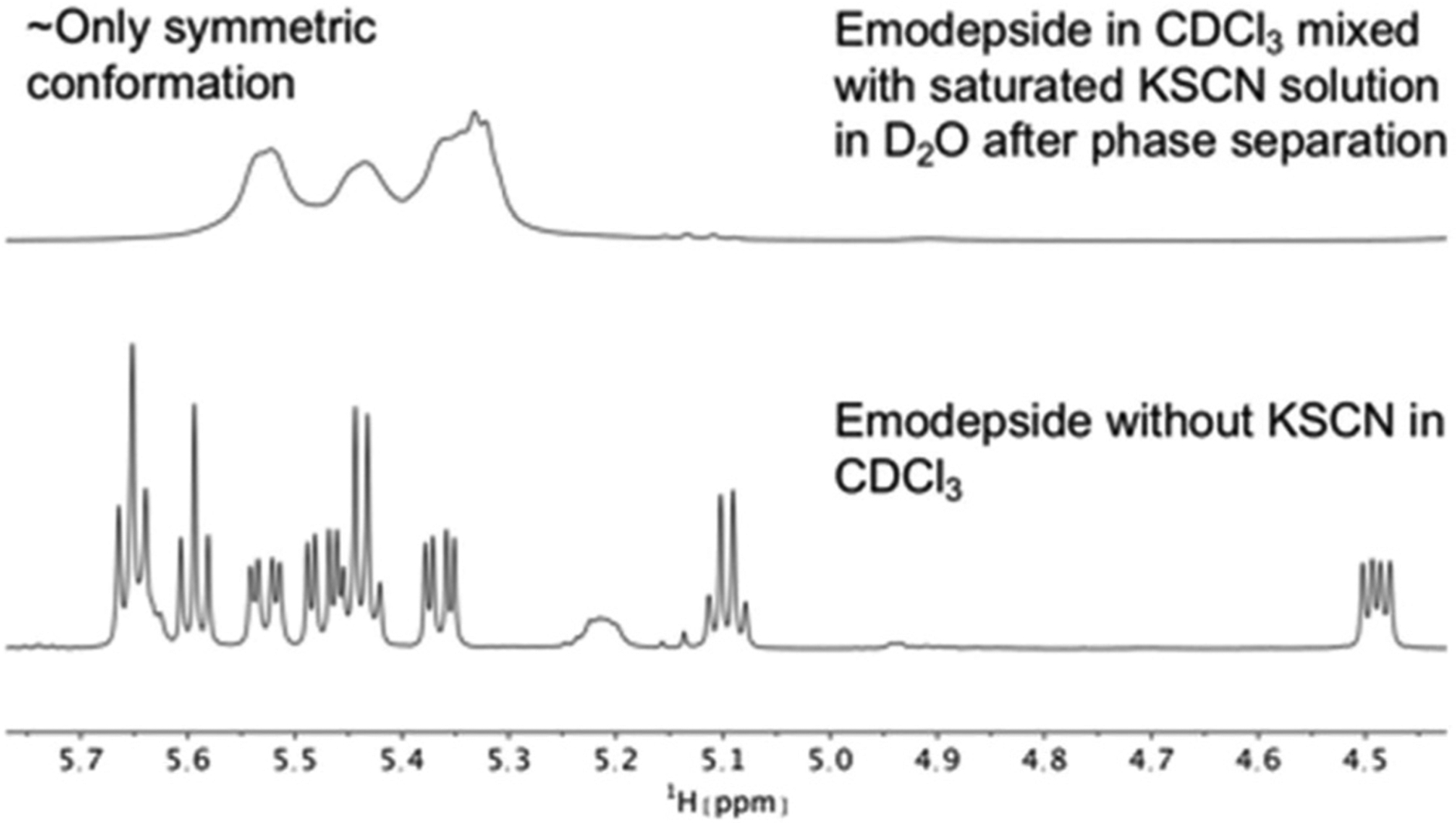
Comparison of ^1^H NMR spectra of the Hα region of emodepside (**2**) in CDCl_3_ measured on a 600 MHz spectrometer. After mixing with a saturated KSCN solution in D_2_O, followed by sonication and phase separation, the symmetric conformation is present almost exclusively in solution.

The same effect was achieved by mixing a solution of emodepside (**2**) in chloroform with a saturated aqueous KSCN solution and letting the solution stand until phase separation had occurred (Fig. 8). These results demonstrate that PF1022A and emodepside can carry cations from a polar phase into an apolar environment.

#### Characterization of the ion-bound complex structure.

**Fig. 9:**
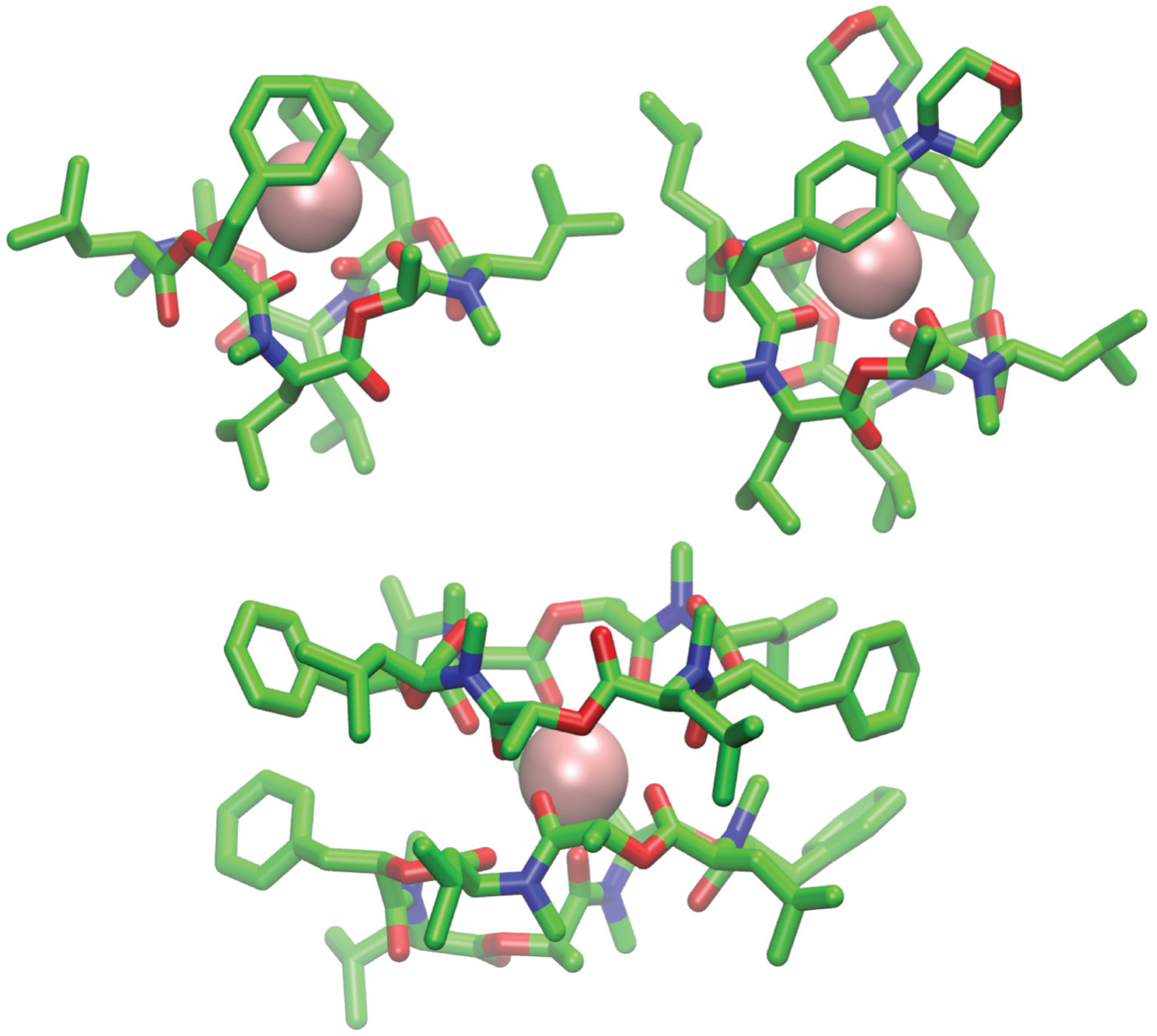
(Top): Snapshot of the 1 : 1 complex from the MD simulation of a single molecule of **1** (left) and **2** (right) in chloroform in presence of a single potassium ion (pink). Both depsipeptides adopt a cavity-like conformation with the cation bound in the center. The same structure could be observed for **1** in methanol after longer simulation time. (Bottom): Snapshot of the 2 : 1 complex from the MD simulation of two molecules of **1** in chloroform in presence of a single potassium ion. Carbons are shown in green, nitrogen atoms in blue, oxygen atoms in red and potassium ions in pink The figures were generated with VMD.^62^

The possible structure of the depsipeptide-ion complex was first investigated *in silico*. MD simulations in presence of a K^+^ ion starting from the symmetric crystal structure for **1** and **2** in methanol (10 μs) and chloroform (1 μs) as well as an MD simulation starting from the asymmetric crystal structure for **1** in chloroform showed that the ion binds to the peptide in the symmetric conformation independent of the starting structure, as expected from the experiment. Furthermore, a cavitand-like structure was adopted, in which the four amide oxygens and the two phenyl rings interact with the cation (Fig. 9). In this highly symmetric conformation, the polar groups are saturated by the metal ion, whereas the side chains of the *N*-methyl leucine residues shield them against the apolar environment.

The ion-bound conformation in the MD simulations is, however, dependent on the system setup. In simulations with two molecules of PF1022A (**1**) in chloroform (1 μs) in presence of a single potassium ion, both a 1 : 1 and a 2 : 1 complex (Fig. 9) could be observed over the course of the simulation, whereby the 1 : 1 complex did not adopt a cavitand-like structure.

**Fig. 10:**
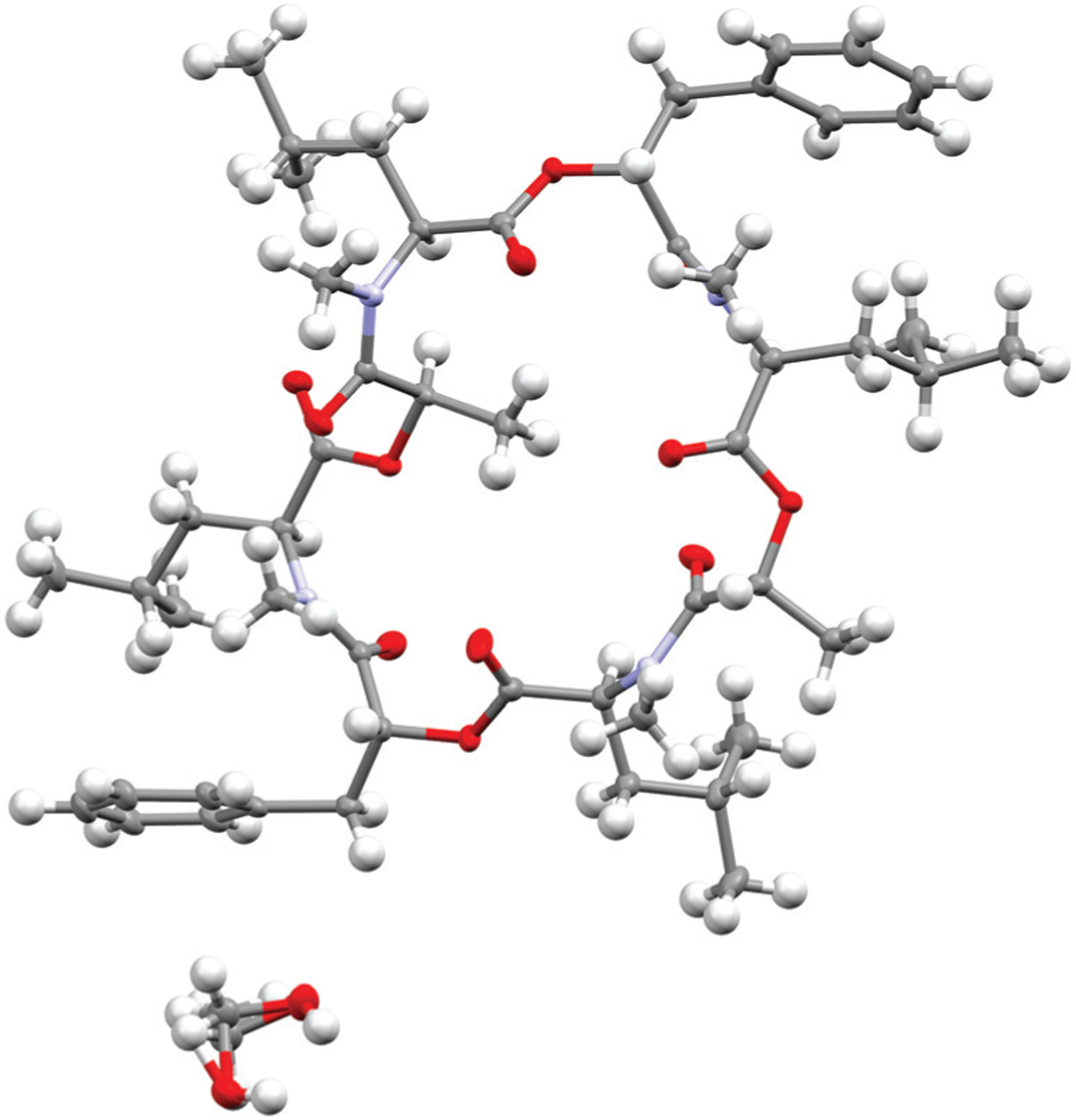
Crystal structure of PF1022A (**1**) (CCDC number: 2004078†) crystallized in the asymmetric conformation. Carbon atoms are colored in grey, nitrogen atoms in light blue and oxygen in red. The ellipsoids represent 50% of probability level and hydrogen atoms are shown with a radius of 0.3 Å. One methanol molecule is co-crystalized and disordered. The figure was created with Mercury.^63^

**Fig. 11:**
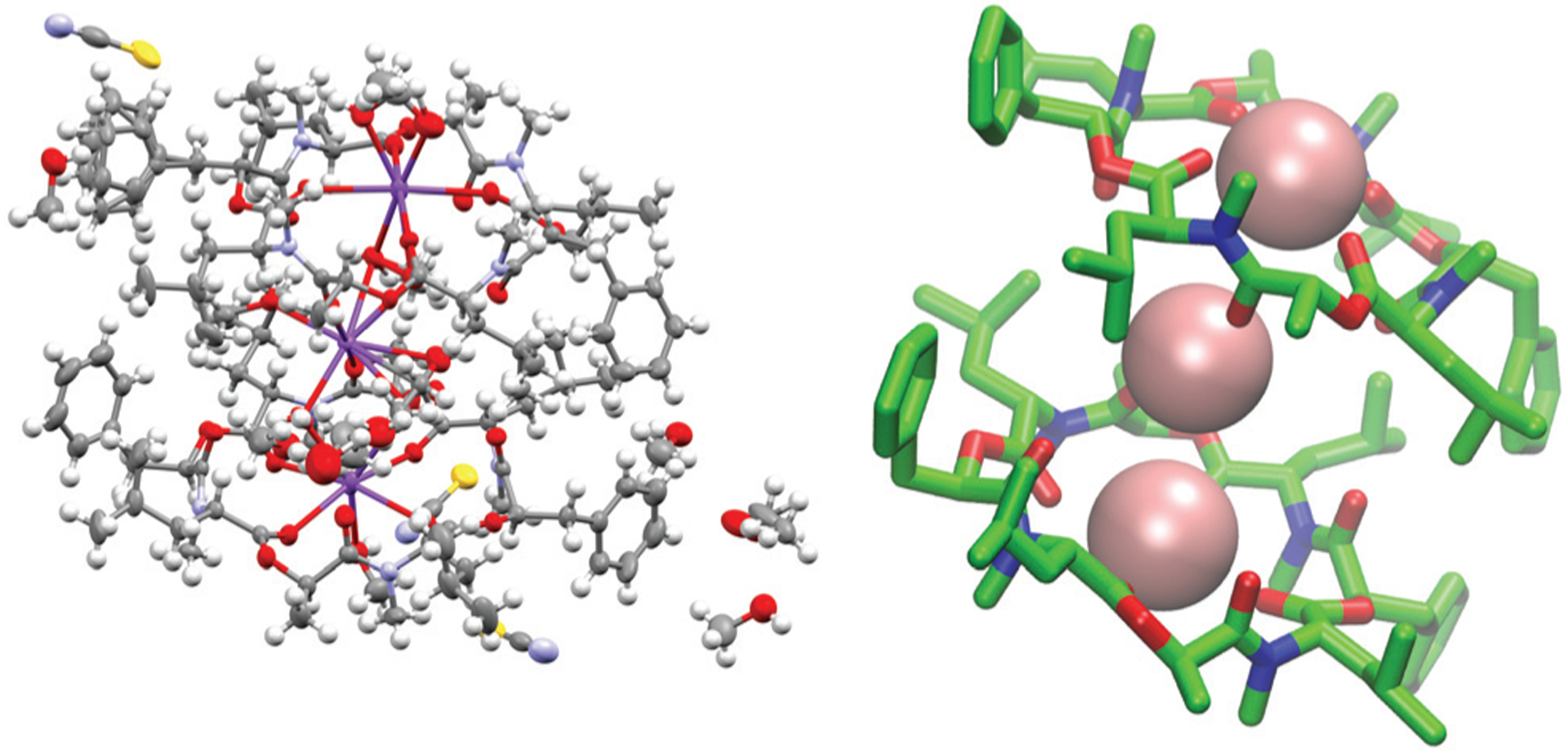
(Left): Crystal structure of a 2 : 3 complex of PF1022A (**1**) with KSCN (CCDC number: 2004087†). There are three potassium ions (purple) crystalized with two molecules of the peptide. Carbon atoms are depicted in grey, nitrogen atoms in light blue, oxygen atoms in red, sulphur atoms in yellow and hydrogen atoms in white. The ellipsoids represent 50% of probability level and hydrogen atoms are shown with a radius of 0.3 Å. One water molecule is co crystalized as well as some methanol. The figure was generated with Mercury.^63^ (Right): Simplified complex structure with only the non-hydrogen atoms present and without co-crystallized solvent molecules. Carbons are shown in green, nitrogen atoms in blue, oxygen atoms in red and potassium ions in pink. The figure was generated with VMD.^62^

To verify the cavitand-like structure of the 1 : 1 complex experimentally, we first aimed to crystalize PF1022A (**1**) in the presence of KSCN. Crystallization attempts with equimolar salt and peptide concentration led to separate crystals of KSCN and **1**, in which **1** is crystallized in the asymmetric conformation with one co-crystallized methanol molecule (Fig. 10). The structure agrees well with the asymmetric crystal structure of the bis-aza analog (**8**) (CCDC code QOXDOW), justifying the use of the latter as starting structure in the MD simulations of **1**. By increasing the KSCN concentration to a 10-fold excess in methanol, an ion-bound complex of **1** could be crystallized. The crystal structure revealed a 2 : 3 complex (peptide : cation), with co-crystallized methanol and one water molecule (Fig. 11). The ion-bound peptide crystallized in the symmetric conformation as observed in the NMR experiments and the MD simulations. This complex is likely not the major structure present in solution. In an MD simulation, the 2 : 3 complex showed very low stability.

**Fig. 12:**
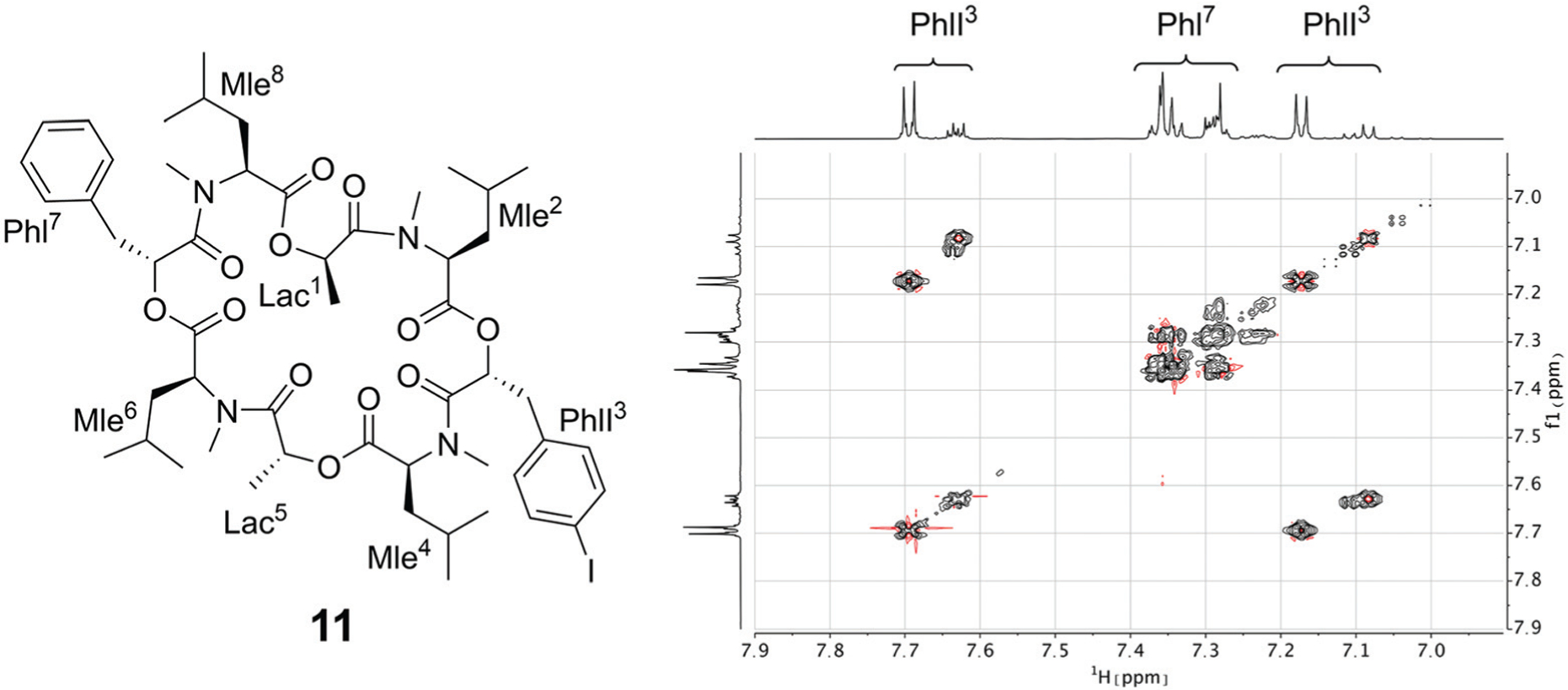
(Left): Chemical structure of the mono-iodine PF1022A analog **11**. (Right): EASY-ROESY spectrum of the aromatic region of 5 mM of **11** with 125 mM CsSCN in CD_3_OH at room temperature with a mixing time of 700 ms. Only correlations within the aromatic rings were observed but no correlation between them.

Since the crystallization experiments were not able to confirm the cavitand-like structure, we next turned to NMR to answer this question. The most straightforward evidence would be a through-space correlation between the two aromatic rings, which should be very close in the cavitand-like structure. However, this correlation is not experimentally accessible in these cyclic depsipeptides due to the *C*_2_ symmetry of the symmetric conformer. One possible solution for this issue is to break the *C*_2_ symmetry by introducing a substitution in the aromatic ring of one of the two phenyllactic acids. The PF1022A analog **11** contains an iodine substituent in *para*-position at one of the aromatic rings (Fig. 12), and exhibits the same conformational behavior and ionophoric properties as **1** (experimental results summarized in the ESI†). With **11**, it should be possible to observe ROESY correlations between the two aromatic rings, if the cavitand-like structure is present in solution. However, such correlations were not observed (Fig. 12). Therefore, the cavitand-like structure is likely an artifact of the setup in the MD simulation with a single peptide and potassium ion. This is further supported by the observation that no cavitand-like structure was adopted in the MD simulations with two peptides and a potassium ion (see discussion above).

### Connection with membrane permeability

Some members of the subfamily of cyclic depsipeptides with all backbone amides methylated have shown decent permeability in parallel artificial membrane permeability assays (PAMPA), *e.g.* for enniatin B (**5**) a log *P*_e_ value of −4.73 was determined.^18^ For PF1022A (**1**) or emodepside (**2**), no permeability data has been reported in the literature. To assess whether the macrocyclic core conformational preference (**1** and **2** *versus* **8**) and the ionophoric property of the cyclic octadepsipeptides influence the passive permeability, PAMPA measurements with and without potassium salt were performed using a protocol similar to that employed for enniatin B (**5**).^18^ Surprisingly, no permeability was detected for PF1022A (**1**) and the related compounds (**2**, **8**) independent of the addition of potassium salt (see Table S12 in the ESI†). These results suggest that the investigated depsipeptides may not permeate but rather incorporate into the membrane (potentially bound to a cation in a 2 : 1 or 1 : 1 complex). Membrane incorporation would agree with the current hypothesis of the mode of action of emodepside, since SLO-1 and the latrophilin receptor are both associated with the membrane.^41^ In addition, it was reported during electrophysical studies that washout of PF1022A incorporated in membranes of CaCo-2 cells was not effective, indicating permanent incorporation into the membrane.^56^ It would also not contradict the observation that emodepside is a substrate of the efflux transporter P-gp,^64^ for which also a membrane-mediated mechanism is proposed.^65^

## Conclusions

In this work, we investigated the conformational behavior and ionophoric property of PF1022A (**1**), emodepside (**2**), and related compounds using NMR experiments and extensive MD simulations in order to establish a connection between them and potentially the membrane permeability. In support of previous literature, two major macrocyclic core conformers were detected in NMR measurements in chloroform and methanol, the major one with one amide bond in *cis*-configuration (asymmetric conformation) and the minor one with all *trans*-amide bonds (symmetric conformation). The symmetric core conformation showed a higher flexibility on the microsecond to millisecond time scale compared to the asymmetric one both in NMR (*i.e.* shorter *T*_2_ relaxation times due to additional exchange contribution) and in kinetic models constructed from the MD data.

Upon addition of cations, a shift towards the symmetric conformation was observed in the NMR titration experiments, which indicates that only the symmetric conformation can bind tightly to the ions. A preference for cesium over potassium over sodium was found, which is in agreement with previous studies. Furthermore, we could show that these cyclic octadepsipeptides can carry cations into an apolar solvent, like other ionophores. The titration curves indicate a mixture of both 1 : 1 and 2 : 1 (2 peptides and 1 cation) complexes. MD simulations suggest the formation of a sandwich complex, like the one observed for enniatin B (**5**). A cavitand-like structure of the 1 : 1 complex seen in the MD simulations could, however, not be confirmed experimentally using the mono-iodine substituted analog (**11**). Crystallization of PF1022A (**1**) with an excess of KSCN in methanol yielded a 2 : 3 complex (2 peptides and 3 potassium ions), where the peptides are in the symmetric conformation, confirming the findings in the NMR experiments and MD simulations.

The fact that the symmetric conformers can bind cations might still be relevant for activity, since the metal bound species may possess a higher propensity for membrane incorporation than the free peptide. This would also be in line with the location of the proposed target, SLO-1, a membrane-bound ion channel. The results of the PAMPA experiments and the ineffective wash-out of PF1022A from CaCo-2 membranes may indeed indicate that the peptides do not permeate but rather incorporate into the membrane. Our extensive NMR and computational characterizations are in this case very important to provide further insight at atomic resolution beyond the scope of PAMPA. In terms of the investigated properties, no significant differences were found between **1** and **2**. The ratios between symmetric and asymmetric conformations in solutions as well as their binding affinities towards cations are similar. Thus, the difference in anthelmintic activity between **1** and **2** cannot be directly related to a difference in the conformational behavior or ionophoric property, but likely stems from the effect of the morpholino substitution modulating the potency at the target. The studied bis-aza analog (**8**), for which the asymmetric conformation is further stabilized, has a significantly lower affinity towards cations, which could be an indication that cation binding may be an important aspect for membrane incorporation, and potentially influence activity. Future studies with cyclic octadepsipeptides that exhibit different cation binding affinities might be able to further elucidate these connections.

## Experimental section

### Peptide synthesis

The methods for obtaining the depsipeptides investigated in this work have been previously reported in the literature.^5,36,66^

### NMR characterization of PF1022A (1), emodepside (2), bis-aza PF1022A analog (8) and mono-iodo analog (11) in CD_3_OH and CDCl_3_

20 mM solutions of **1** (12.3 mg), **2** (14.6 mg), **8** (12.4 mg) and **11** (14.0 mg) in methanol-d_3_ (Armar) as well as in chloroformd (Cambridge Isotope Laboratories) were used for the characterization by NMR. Because of solubility issues of **2** in methanol, a 6.7 mM solution was used instead (4.4 mg). A full set of spectra (^1^H-NMR, ^13^C-NMR, TOCSY, double-quantum filtered COSY, multiplicity edited ^13^C-HSQC with adiabatic decoupling, ^13^C-HMBC, ^15^N-HMBC and EASY-ROESY^67^) was recorded for each compound except for **11** where no ^13^C-NMR spectrum was recorded. If not stated otherwise all spectra were measured at 25 °C on a Bruker Avance III HD 600 MHz spectrometer equipped with a N_2_-cooled Prodigy triple resonance probe with *z*-gradients or on a Bruker AVANCE III 500 MHz spectrometer equipped with a BBFO broadband probe with *z*-gradients.

The CD_3_OH signal was suppressed by presaturation or excitation sculpting.^68 13^C-HSQC spectra were recorded with sensitivity enhancement^69^ and multiplicity editing. TOCSY spectra were recorded with zero quantum filter^70^ and 80 ms DIPSI2^71^ isotropic mixing except for **1** in chloroform where 80 ms mlev17^72^ mixing was used. The mixing time for the EASY-ROESY experiments was set to 100 ms if not otherwise stated. For all 2D spectra, the time domain in both dimensions was extended to twice its size by zero filling and apodized with a cos^2^ or sin function. The baseline of the resulting spectra was corrected with a polynomial of fifth order or using the Whittacker smoother algorithm.^73^ Processing was done with Bruker TopSpin™ version 4.0 (Bruker Biospin AG) and MestReNova 12.0 (Mestrelab Research). Resonance assignment and integration of ROESY cross-peaks were performed with SPARKY 3.115.^74 13^C *T*_2_-relaxation time measurements were done with a series of sensitivity enhanced ^13^C-CPMG-HSQC spectra^75^ using a slightly modified version of Bruker standard pulse program hsqct2etf2gpsi with ten different evenly spaced relaxation delays between 15.2 ms and 456 ms. Heating effect compensation was used. Fitting of the exponential decays was done with Prism 8.4 (GraphPad Software).

### Calculation of exchange rates

Following ref. 76 and 77, site-to-site rates were determined in a straightforward way by taking the logarithm of matrix *A* = *M* × *M*_0_^−1^ containing the volumes of cross- and diagonal peaks of the exchanging sites (Hα of Mle^2^, Mle^6^ and Mle^26^) divided by their magnetic fraction *M*_0_ (*i.e.* the relative intensities of the corresponding resonances in the ^1^H NMR spectrum) as an approximation of *M*(0):

(1) $$M=e^{{\text{Lt}}_{\text{m}}}\times M_{0}$$

(2) $$A=\left(\begin{array}{ccc} \frac{I_{\text{AA}}}{M_{\text{0A}}} & \frac{I_{\text{AB}}}{M_{\text{0B}}} & \frac{I_{\text{AC}}}{M_{\text{0C}}}\\ \frac{I_{\text{BA}}}{M_{\text{0A}}} & \frac{I_{\text{BB}}}{M_{\text{0B}}} & \frac{I_{\text{BC}}}{M_{\text{0C}}}\\ \frac{I_{\text{CA}}}{M_{\text{0A}}} & \frac{I_{\text{CB}}}{M_{\text{0B}}} & \frac{I_{\text{CC}}}{M_{\text{0C}}} \end{array}\right)$$

(3) $$\frac{1}{t_{\text{m}}}\text{ln}(A)=\left(\begin{array}{lll} k_{\text{BA}}+k_{\text{CA}}-R_{1} & k_{\text{AB}} & k_{\text{AC}}\\ k_{\text{BA}} & -k_{\text{AB}}-R_{1} & 0\\ k_{\text{BC}} & 0 & -k_{\text{AC}}-R_{1} \end{array}\right)$$

*L* is the difference between the kinetic matrix *K* containing the site-to-site rate constants and the relaxation matrix *R*, *t*_m_ is the mixing time used in the ROESY experiment, and *R*_i_ are the auto-relaxation rates of the exchanging sites in the symmetric (A) and asymmetric (B, C) conformations.

As an example, the procedure is described in the following for PF1022A (**1**).

**Fig. 13:**
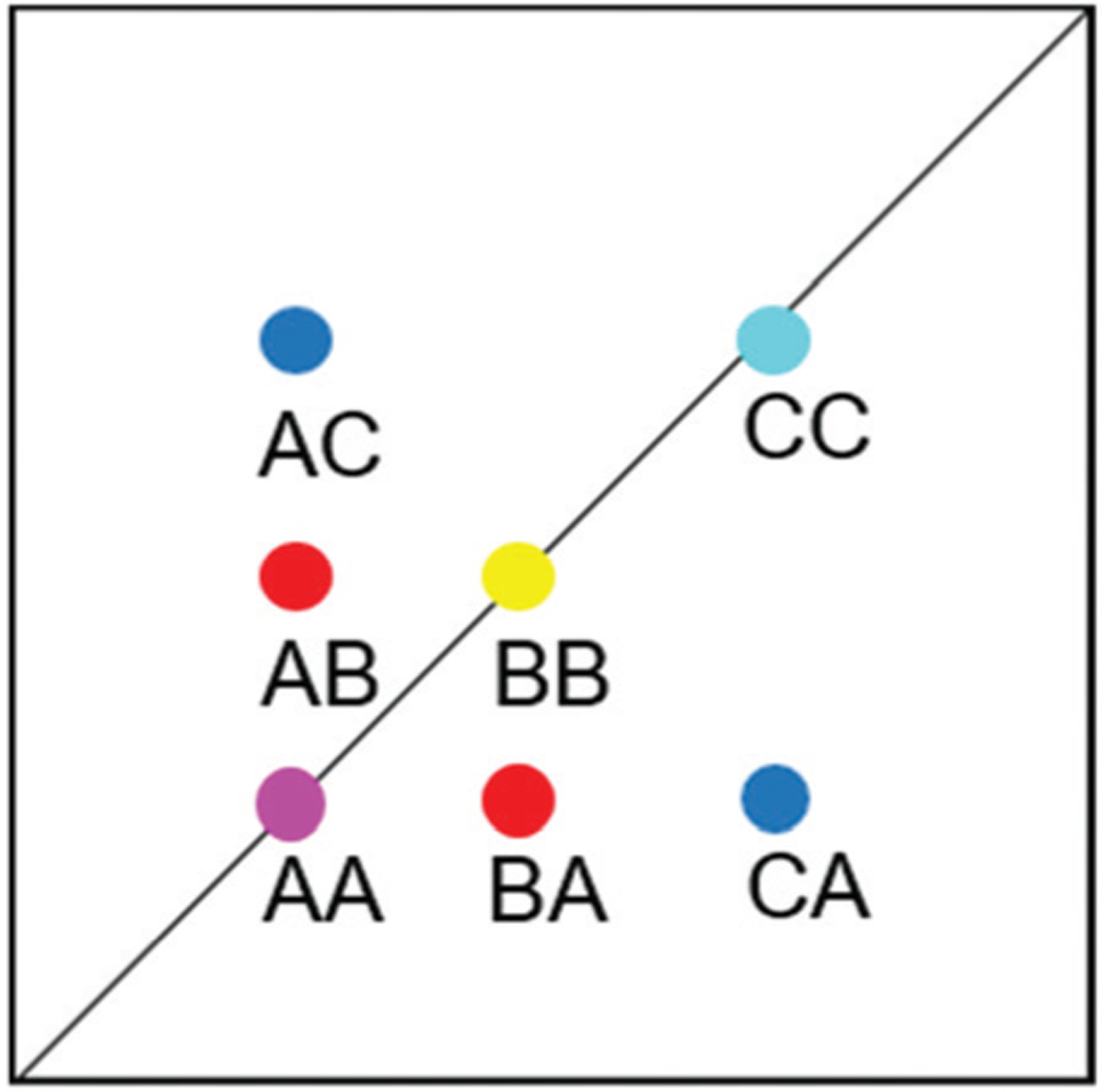
Schematic EXSY spectrum with sites A (symmetric conformation), B and C (asymmetric conformation). A exchanges with B and C but B does not exchange with C.

EXSY peak volumes extracted from the ROESY spectrum (shown schematically in Fig. 13) and the magnetic fractions in the 1H NMR spectrum are inserted in matrix *A* (eqn 4). Site-to-site rates are obtained by taking the logarithm of matrix *A* and dividing the result by the mixing time (0.1 s) (eqn 5): *k*_AB_ = *k*_AC_ = 0.09 s^−1^ and *k*_BA_ = *k*_CA_ = 0.16 s^−1^ (averaged rates). Calculations were carried out in Mathematica 12.0.^78^

(4) $$A=\left(\begin{array}{ccc} \frac{77.9}{1} & \frac{1.52}{1.5} & \frac{1.55}{1.5}\\ \frac{1.59}{1} & \frac{236}{1.5} & \frac{0}{1.5}\\ \frac{1.75}{1} & \frac{0}{1.5} & \frac{200}{1.5} \end{array}\right)$$

(5) $$\frac{1}{0.1}\text{ln}(A)=\left(\begin{array}{lll} 43.5524 & 0.09 & 0.10\\ 0.14 & 50.58 & 0.00\\ 0.17 & 0.00 & 48.9 \end{array}\right)$$

### Titration with monovalent cations

CsSCN was prepared by dissolving Cs_2_CO_3_ (100 mg, 0.31 mmol, Sigma-Aldrich) and NH_4_SCN (46.7 mg, 0.62 mmol, Sigma-Aldrich) in 0.5 ml water and was then crystalized at room temperature.^79^ The crystals were washed with cold water and then dried in the oven at 105 °C. Aliquots of a 100 mM and a 1 M solution of KSCN (Fluka), NaSCN (Sigma-Aldrich) and NH_4_SCN (Merck) and a 100 mM solution of CsSCN in methanol-d_3_ were used to titrate a 5 mM solution of **1** and a 5 mM solution of **2** (only with KSCN and NaSCN) as well as a 5 mM solution of **8** (only with KSCN) and **11** (only with CsSCN). For compound **1**, ^1^H spectra with solvent suppression using excitation sculpting were recorded at 0, 2.5, 5, 10, 20, 50, 100 and 200 mM KSCN. For the other titrations, 1D-NOESY spectra with presaturation (mixing time 10 ms) were recorded as it was observed that the intensities near the solvent signal were affected by the excitation sculpting. In addition, the base lines were flatter in the 1D-NOESY spectra, which was more favorable for integration of the peak intensities. Spectra were recorded at salt concentrations of 0, 0.25, 0.5, 1, 2, 5, 10, 20, 50, 100, and 200 mM, except for CsSCN, where the maximal concentration was 125 mM. For titrations of **1** with KSCN, additional data points at 75, 125, 150, and 175 mM were recorded. For titrations of **1** and **11** with CsSCN, additional data points at 75 and 125 mM were recorded.

### PAMPA measurements

PAMPA was performed using a 96-well filter donor plate with 0.45 μm hydrophobic Immobilon-P membrane supports (Millipore MAIPNTR10) and a 96-well Teflon acceptor plate (Millipore MSSACCEPTOR). The donor plate was prepared by applying 5 μL of 1% (w/v) soybean lecithin (90%, Alfa Aesar) in *n*-dodecane to each filter. The acceptor plate was prepared by addition of 300 μl of pH = 7.4 phosphate-buffered saline (PBS) with 5% DMSO to each well. Donor solutions composed of PBS with 5% DMSO, 10 μM carbamazepine standard, and 10 μM compound **1**, **2** or **8** were prepared with and without additional KCl (0.27 mM or 100.27 mM K^+^ concentration) before addition of 150 μl to each donor well, with all conditions run in quadruplicate. The donor plate was then loaded into the acceptor plate and was left in a sealed, damp chamber at 25 °C for approximately 17 hours (exact times recorded and used in calculation of *P*_e_). The plates were then separated, and 100 μl of solution from each well was transferred to another plate for LC-MS analysis. Permeabilities were calculated identically to a previous study.^80^

### MD simulations

The GROMOS simulation package^45^ was used for all simulations together with the GROMOS 54A7 united-atom force field^46^ for the solvent and the peptides, and the 2016H66 force field^81^ for the potassium ion. MD simulations of 1–10 μs length were performed under isothermal-isobaric conditions (NPT) using the leap-frog integration scheme with a time step of 2 fs.^82^ The temperature was kept at 298 K by weak coupling to two separate temperature baths for the peptide and the solvent with a relaxation time of 0.1 ps and the pressure was kept at 1 atm by weak coupling to a pressure bath with a relaxation time of 0.5 ps and an isothermal compressibility of 4.5 × 10^−4^ kJ^−1^ mol nm^3^.^83^ A twin range cutoff scheme was used with cutoffs of 0.8 and 1.4 nm for the non-bonded interactions. Bond lengths were constrained with the SHAKE algorithm with a tolerance of 10^−4^ nm (ref. 84) and center of mass motion was removed every 1000 steps. MD simulations were performed in chloroform and methanol using dielectric permittivity coefficients taken from ref. 85 for the dielectric continuum outside the cutoff (reaction-field method).^86^ Systems with K^+^ ions were simulated without counter-charge because the two ions would aggregate in chloroform and methanol. The GROMOS++ program “ion” was used to replace the solvent molecule with the lowest electrostatic potential energy by a potassium ion.^87^ The crystal structure of emodepside (**2**) (CCDC code DOMZOW)^35^ was used as the symmetric starting structure, and the crystal structure of the bis-aza analog (**8**) as the asymmetric starting structure (CCDC code QOXDOW).^36^ The peptides were minimized first in vacuum using a steepestdecent algorithm.^88^ The peptide was solvated in the corresponding solvent and the solvent was relaxed while the coordinates of the peptide were restrained with a force constant of 2.5 × 10^4^ kJ mol^−1^ nm^−2^. Afterwards, the system was thermalized to 298 K in five steps of 60 K and the force constant was loosened one order of magnitude in each step if not otherwise stated. Initial velocities were generated using a Maxwell-Boltzmann distribution. Details of the performed simulations are summarized in Table 4.

### Markov state model (MSM) building

**Fig. 14:**
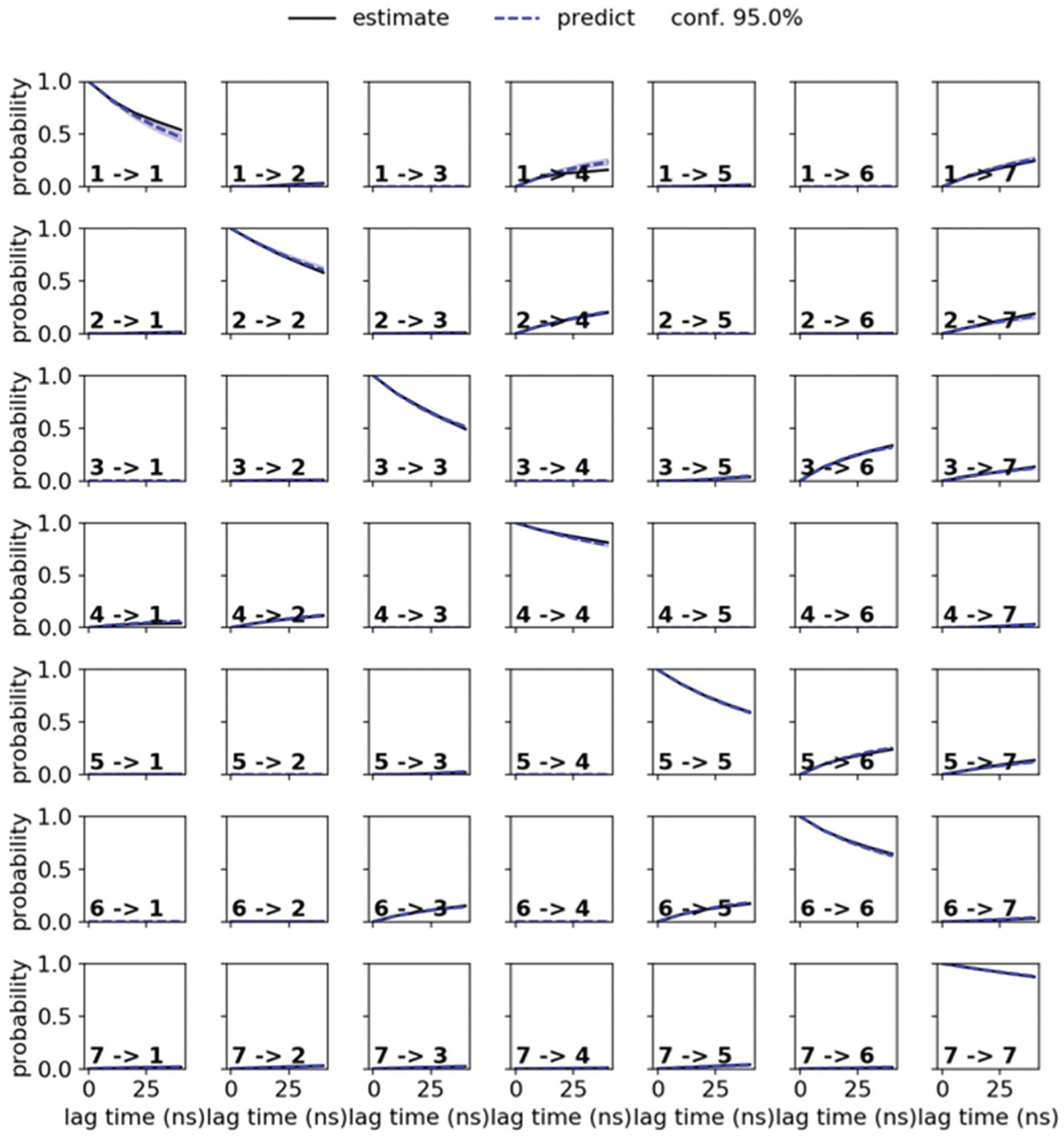
Chapman-Kolmogorov test for the symmetric conformer of **1** in chloroform with 7 states and a lag time of 10 ns.

MSMs were built using the PyEMMA package.^54^ Ten 1 μs and one 10 μs MD simulations starting from the symmetric and from the asymmetric crystal structure were used to build the MSM in chloroform for PF1022A (**1**). Input features were all backbone dihedrals. Time-lagged independent component analysis (TICA)^55^ was done with a lag time of 10 ns. A common nearest neighbor (CNN) density based clustering^49^ with a similarity of 10 and a cutoff distance of 0.15 was applied.^53^ 20% of the input data was discarded as noise. The regions with asymmetric and symmetric conformations were not connected, since the *trans*-to-*cis* transition of the pertinent amide bond was never sampled. The asymmetric set consisted of two non-connected subsets. One of them arose from a single simulation and was therefore discarded as noise. Implied timescales from a Bayesian MSM revealed six slow processes for the symmetric conformer. Chapman-Kolmogorov test^52^ (Fig. 14) was used to validate the model with seven conformational states. Finally, an MSM was constructed for these seven states (see main text and Fig. S6 in the ESI†).

### Crystallization of 1 with KSCN in methanol

Around 10 mg of **1** was dissolved in methanol together with an equimolar amount of KSCN (1.0 mg). The sample was put in the freezer at −28 °C. After five days, transparent crystals were obtained. Analysis was done by the small molecules crystallography center (SMOCC) at ETH Zürich. A XtaLAB Synergy, Dualflex, Pilatus 300 K diffractometer was used for both measurements. The crystal was kept at 100 K during data collection. Using Olex2,^89^ the structure was solved with the ShelXT^90^ structure solution program using Intrinsic Phasing and refined with the ShelXL^91^ refinement package using Least Squares minimization. The obtained crystal structure was only the peptide without the salt in its asymmetric form. **Crystal data** for C_53_H_80_N_4_O_13_ (*M* = 981.21 g mol^−1^): monoclinic, space group *P*2_1_ (no. 4), *a* = 14.40860(10) Å, *b* = 13.78330(10) Å, *c* = 14.46940(10) Å, *β* = 110.1570(10)°, *V* = 2697.59(4) Å^3^, *Z* = 2, *T* = 100.0(1) K, *μ*(Cu Kα) = 0.701 mm^−1^, *D*_calc_ = 1.208 g cm^−3^, 74 402 reflections measured (6.508° ≤ 2*Θ* ≤ 159.456°), 11 334 unique (*R*_int_ = 0.0423, *R*_sigma_ = 0.0233) which were used in all calculations. The final *R*_1_ was 0.0280 (*I* > 2*σ*(*I*)) and w*R*_2_ was 0.0699 (all data).

Around 10 mg of **1** was dissolved in methanol together with a tenfold excess of KSCN. The concentrated sample was put in the freezer at −28 °C. After three days transparent crystals were obtained and were given to SMOCC for analysis. The obtained crystal structure was a complex of two peptides with three ions with co-crystalized methanol molecules and one water molecule. **Crystal data** for C_118_H_198_K_3_N_11_O_36_S_3_ (*M* = 2560.34 g mol^−1^): triclinic, space group *P*1 (no. 1), *a* = 14.90080(10) Å, *b* = 15.83070(10) Å, *c* = 16.84690(10) Å, *α* = 111.2440(10)°, *β* = 101.4290(10)°, *γ* = 100.0950(10)°, *V* = 3495.22(5) Å^3^, *Z* = 1, *T* = 100.0(1) K, *μ*(CuKα) = 1.908 mm^−1^, *D*_calc_ = 1.216 g cm^−3^, 95 494 reflections measured (5.872° ≤ 2*Θ* ≤ 159.716°), 28 018 unique (*R*_int_ = 0.0424, *R*sigma = 0.0371) which were used in all calculations. The final *R*_1_ was 0.0502 (*I* > 2*σ*(*I*)) and w*R*_2_ was 0.1453 (all data).

## Supplementary Material

Main SI

CIF file

## Figures and Tables

**Table 1:** Ratios between asymmetric and symmetric conformer in CD_3_OH and CDCl_3_ for compounds **1**, **2** and **8**. Literature values are given in parentheses. Ratio marked with * was reported in CD_3_OD

Compound	Conformer ratio in CD_3_OH (asymmetric : symmetric)	Conformer ratio in CDCl_3_ (asymmetric : symmetric)
PF1022A (**1**)	5 : 1–7 : 1^a^ (4 : 1*^9^)	3 : 1 (3 : 1^38^)
Emodepside (**2**)	7 : 1	7 : 2
Bis-aza analog (**8**)	12 : 1	10 : 1 (100 : 7^36^)

a The variability is likely due to residual cation content originating from synthesis, workup and purification that differs from batch to batch.

**Table 2:** Site-to-site exchange rates between asymmetric and symmetric conformers measured in EASY-ROESY experiments with mixing time of 100 ms in CDCl_3_

Compound	*k*_1_ [s^−1^]	*k*_2_ [s^−1^]	*k*_ex_[s^−1^]
PF1022A (**1**)	0.16	0.09	0.25
Emodepside (**2**)	0.12	0.06	0.18
Bis-aza analog (**8**)	0.17	0.02	0.19

**Table 3:** Change in ratio between asymmetric and symmetric conformers without salt and after addition of a 40-fold excess of the salt (25-fold in case of CsSCN due to solubility issues) in CD_3_OH

Salt	PF1022A (1)	Emodepside (2)	Bis-aza analog (8)
KSCN	7 : 1 to 1 : 17	7 : 1 to 1 : 15	12 : 1 to 1 : 0.8
NaSCN	5 : 1 to 1 : 3	7 : 1 to 1 : 3	—
NH_4_SCN	7 : 1 to 1 : 1	—	—
CsSCN	7 : 1 to 1 : 50	—	—

**Table 4:** Details of the performed MD simulations. Thermalizations marked with * were done with a single step directly at 298 K instead of five steps. Simulations marked with ^#^ were done with modified partial charges for the methylated amides

System	Starting structure (CCDC code)	Number of simulations	Solvent	Number of solvents	Length of thermalization per step [ps]	Length per MD simulation [μs]
PF1022A	DOMZOW	1	CHCl_3_	329	2000*	10
PF1022A	DOMZOW	10	CHCl_3_	329	2000	1
PF1022A	DOMZOW	1	CH_3_OH	637	20	1
PF1022A	QOXDOW	1	CHCl_3_	344	2000	10
PF1022A	QOXDOW	10	CHCl_3_	344	2000	1
PF1022A + K^+^	DOMZOW	1	CHCl_3_	328	20	1
PF1022A + K^+^	DOMZOW	1	CH_3_OH	636	20	10
PF1022A + K^+^	QOXDOW	1	CHCl_3_	343	20	1
2 PF1022A + K^+^	DOMZOW	1	CHCl_3_	3085	2000	1
2 PF1022A + 3K^+^	2004087	1	CHCl_3_	389	2000	1
2 PF1022A + 3 K^+^	2004087	1	CH_3_OH	765	2000	1
Emodepside	DOMZOW	1	CHCl_3_	497	2000*	10
Emodepside	DOMZOW	1	CH_3_OH	989	20	1
Emodepside + K^+^	DOMZOW	1	CHCl_3_	496	20	1
Emodepside + K^+^	DOMZOW	1	CH_3_OH	988	20	10
Bis-aza analog	DOMZOW	11	CHCl_3_	330	2000	1
Bis-aza analog	QOXDOW	1	CHCl_3_	350	2000*	10
Bis-aza analog	QOXDOW	10	CHCl_3_	350	2000	1
Bis-aza analog	QOXDOW	1	CH_3_OH	688	20	1
Bis-aza analog + K^+^	QOXDOW	1	CHCl_3_	349	20	10
Bis-aza analog + K^+^	QOXDOW	1	CH_3_OH	688	20	1
PF1022A^#^	DOMZOW	1	CHCl_3_	329	2000*	10
PF1022A^#^	DOMZOW	10	CHCl_3_	329	2000*	1
PF1022A^#^	QOXDOW	1	CHCl_3_	344	2000*	10
PF1022A^#^	QOXDOW	10	CHCl_3_	344	2000*	1

## Footnotes

† Electronic supplementary information (ESI) available. CCDC 2004078 and 2004087. For ESI and crystallographic data in CIF or other electronic format see DOI: 10.1039/d0ob01447h
