# Supplementary material for: Connecting the conformational behavior of cyclic octadepsipeptides with their ionophoric property and membrane permeability
Source: Org Biomol Chem. Author manuscript; Available in PMC 2021 Jan 9. (PMC7796559; doi:10.1039/d0ob01447h)
Supplement: Main SI [file NIHMS1659706-supplement-Main_SI.pdf]

Electronic Supplementary Material (ESI) for Organic & Biomolecular Chemistry.
This journal is © The Royal Society of Chemistry 2020

Supporting Information

Connecting the Conformational Behavior of Cyclic Octadepsipeptides With Their Ionophoric Property and Membrane Permeability

Thomas Stadelmann[a], Govindan Subramanian [b], Sanjay Menon[b], Chad E. Townsend[c], R. Scott Lokey[c], Marc-Olivier Ebert*[a], Sereina Riniker*[a]

[a] Department of Chemistry and Applied Biosciences, ETH Zurich, Vladimir-Prelog-Weg 1-5, 8093 Zurich, Switzerland

[b] Veterinary Medicine Research & Development, Zoetis, 333 Portage Street, Bldg. 300, Kalamazoo, Michigan 49007, USA

[c] Department of Chemistry and Biochemistry, University of California, Santa Cruz, California 93064, USA

* marc-olivier.ebert@org.chem.ethz.ch; sriniker@ethz.ch

NMR Assignments:

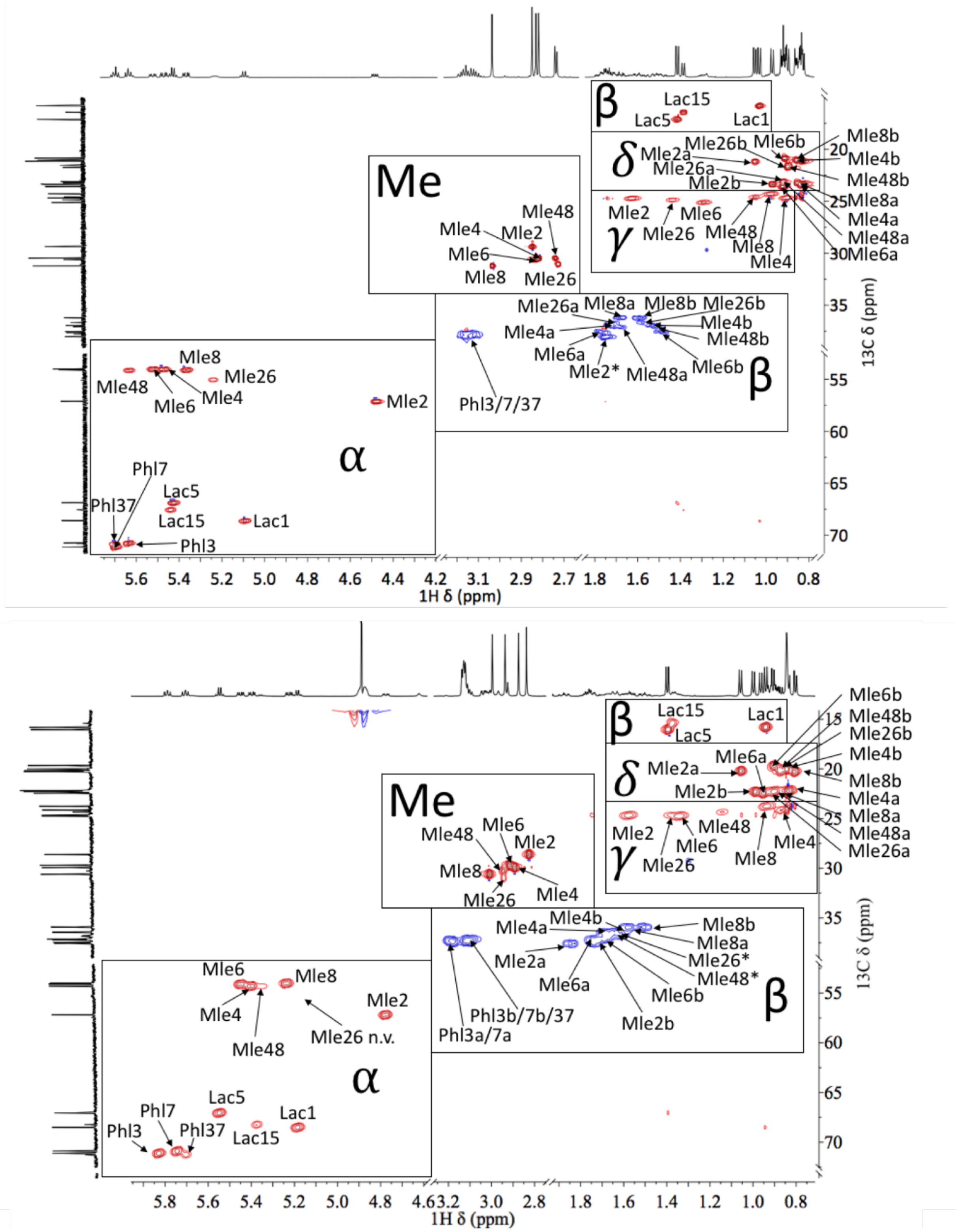

Figure S 1. ^{13}C-HSQC spectra of PF1022A (**1**) in $CDCl_3$ (top) and in CD_3OH (bottom). Empty regions are cut out for clarity. S 7v: not visible with the employed contour levels.

Table S 1. Assignment of the major (asymmetric) and minor (symmetric) conformation of PF1022A (**1**) in $CDCl_3$ referenced to the residual $CHCl_3$ shift in the solvent set to 7.29 ppm. * indicates non-assignable signals due to overlap.

		Hα	Hβ	Hγ	Hδ	Hε	*Hζ*	HMe
Lac^{1}		5.10	1.03	-	-	-	-	-
Mle^{2}		4.49	1.75	1.63	1.05, 0.97	-	-	2.85
Phl^{3}		5.70	3.15	-	7.25	7.31	7.28	-
Mle^{4}		5.47	1.73, 1.53	0.91	0.86, 0.84	-	-	2.82
Lac^{5}		5.43	1.41	-	-	-	-	-
Mle^{6}		5.52	1.77, 1.49	1.29	0.92, 0.91	-	-	2.83
Phl^{7}		5.64	3.17, 3.11	-	7.26	7.30	7.27	-
Mle^{8}		5.37	1.68, 1.60	0.98	0.83, 0.83	-	-	3.04
Lac^{15}		5.44	1.39	-	-	-	-	-
Mle^{26}		5.23	1.72, 1.58	1.44	0.92, 0.90	-	-	2.73
Phl^{37}		5.70	3.16, 3.11	-	7.25-7.35*	7.25-7.35*	7.25-7.35*	-
Mle^{48}		5.63	1.68, 1.50	1.05	0.90, 0.85	-	-	2.74
	C	Cα	Cβ	Cγ	Cδ	Cε	*Cζ*	CMe
Lac^{1}	171.6	68.6	15.8	-	-	-	-	-
Mle^{2}	171.2	57.1	38.0	24.7	21.2, 23.4	-	-	29.4
Phl^{3}	170.0	71.2	37.6	135.1	129.5	128.6	127.2	-
Mle^{4}	169.8	54.0	37.0	24.7	21.0, 23.4	-	-	30.5
Lac^{5}	170.4	66.9	17.1	-	-	-	-	-
Mle^{6}	169.8	54.0	37.5	25.1	23.6, 20.9	-	-	30.6
Phl^{7}	170.2	70.8	38.0	135.4	129.5	128.5	127.1	-
Mle^{8}	171.0	54.1	36.2	24.3	23.4, 21.1	-	-	31.2
Lac^{15}	169.9	67.5	16.4	-	-	-	-	-
Mle^{26}	170.6	55.1	36.7	24.9	23.1, 21.7	-	-	31.1
Phl^{37}	169.4	70.8	37.8	135.4	129.7	128.5	127.0	-
Mle^{48}	170.9	54.1	37.1	24.6	21.6, 23.2	-	-	30.5

Table S 2. Assignment of the major (asymmetric) and minor (symmetric) conformation of PF1022A in CD_3OH referenced to the residual CH_3OH shift in the solvent set to 3.33 ppm. * indicates non-assignable signals due to overlap and n.d. indicates signals that could not be detected.

		Hα	Hβ	Hγ	Hδ	Hε	*Hζ*	HMe
Lac[1]		5.19	0.94	-	-	-	-	-
Mle[2]		4.78	1.85, 1.74	1.58	1.06, 0.99	-	-	2.82
Phl[3]		5.83	3.19, 3.08	-	7.24-7.35	7.24-7.35*	7.24-7.35*	-
Mle[4]		5.40	1.69, 1.64	0.86	0.84	-	-	2.90
Lac[5]		5.55	1.40	-	-	-	-	-
Mle[6]		5.45	1.75, 1.69	1.33	0.95, 0.90	-	-	2.92
Phl[7]		5.75	3.17, 3.11	-	7.24-7.35	7.24-7.35*	7.24-7.35*	-
Mle[8]		5.24	1.58, 1.51	0.94	0.83, 0.80	-	-	3.01
Lac[15]		5.37	1.38	-	-	-	-	-
Mle[26]		5.15	1.61	1.39	0.91, 0.87	-	-	2.94
Phl[37]		5.70	3.10	-	7.32	7.24-7.35*	7.24-7.35*	-
Mle[48]		5.35	1.66	1.14	0.89, 0.87	-	-	2.95
	C	Cα	Cβ	Cγ	Cδ	Cε	*Cζ*	CMe
Lac[1]	173.1	68.5	15.8	-	-	-	-	-
Mle[2]	171.0	57.2	37.6	24.7	20.2, 22.3	-	-	28.6
Phl[3]	171.7	71.1	37.2	134.8	129.4	128.4	126.9	-
Mle[4]	169.3	54.3	36.4	24.1	22.3, 20.0	-	-	29.9
Lac[5]	172.1	67.1	16.1	-	-	-	-	-
Mle[6]	169.6	54.1	37.2	24.8	22.5, 19.7	-	-	29.7
Phl[7]	171.7	70.9	37.5	135.1	129.3	128.3	127.0	-
Mle[8]	170.7	54.0	35.9	23.8	22.2, 20.3	-	-	30.6
Lac[15]	171.9	68.3	15.4	-	-	-	-	-
Mle[26]	n.d.	55.3	36.8	24.6	22.3, 20.1	-	-	31.0
Phl[37]	170.7	71.3	37.1	135.5	129.3	128.3	126.8	-
Mle[48]	170.2	54.3	36.9	24.3	22.2, 20.3	-	-	30.3

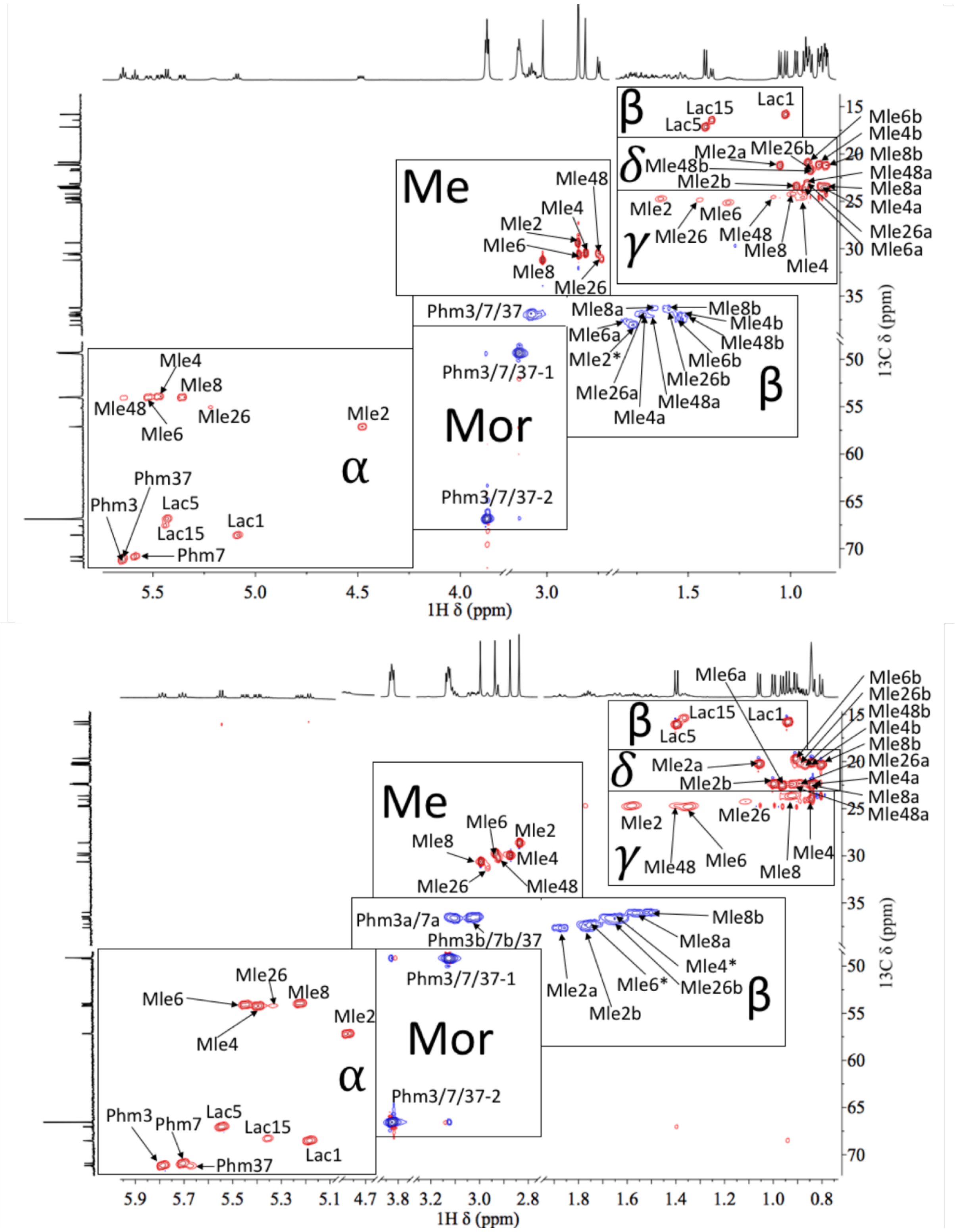

Figure S 2. ^{13}C-HSQC spectra of emodepside (**2**) in $CDCl_3$ (top) and in CD_3OH (bottom). Empty regions are cut out for clarity.

Table S 3. Assignment of the major (asymmetric) and minor (symmetric) conformation of emodepside (**2**) in $CDCl_3$ referenced to the residual $CHCl_3$ shift in the solvent set to 7.28 ppm. * indicates non-assignable signals due to overlap. Mor1 is the C/H next to the oxygen in the morpholine ring, and Mor2 is the C/H next to the nitrogen in the morpholine ring.

		Hα	Hβ	Hγ	Hδ	Hε		HMe	HMor1	HMor2
Lac^{1}		5.09	1.03	-	-	-		-	-	-
Mle^{2}		4.49	1.77	1.64	1.05, 0.98	-		2.85	-	-
Phm^{3}		5.65	3.08	-	7.13-7.17*	6.84		-	3.12-3.16*	3.87
Mle^{4}		5.47	1.73, 1.54	0.94	0.85	-		2.82	-	-
Lac^{5}		5.43	1.42	-	-	-		-	-	-
Mle^{6}		5.53	1.81,1.54	1.31	0.92	-		2.85	-	-
Phm^{7}		5.59	3.07	-	7.13-7.17*	6.85		-	3.12-3.16*	3.87
Mle^{8}		5.36	1.68, 1.60	1.0	0.83	-		3.02	-	-
Lac^{15}		5.44	1.39	-	-	-		-	-	-
Mle^{26}		5.21	1.73, 1.60	1.45	0.92, 0.90	-		2.76	-	-
Phm^{37}		5.64	3.04	-	7.13-7.17*	6.83		-	3.11	3.87
Mle^{48}		5.63	1.69, 1.51	1.09	0.90, 0.86	-		2.74	-	-
	C	Cα	Cβ	Cγ	Cδ	Cε	*Cζ*	CMe	CMor1	CMor2
Lac^{1}	171.7	68.6	15.8	-	-	-	-	-	-	-
Mle^{2}	171.2	57.1	38.1	24.7	21.2, 23.4	-	-	29.4	-	-
Phm^{3}	170.1	71.3	36.8	126.1	130.3	115.6	150.3-150.4*	-	49.3-49.4*	66.9
Mle^{4}	169.8	54.0	37.0	24.6	21.1, 23.6	-	-	30.5	-	-
Lac^{5}	170.3	66.9	17.1	-	-	-	-	-	-	-
Mle^{6}	169.8	54.0	37.6	25.1	20.9, 23.7	-	-	30.6	-	-
Phm^{7}	170.4	70.9	37.1	126.6	130.3	115.6	150.3-150.4*	-	49.3-49.4*	66.9
Mle^{8}	171.1	54.0	36.2	24.2	21.2, 23.5	-	-	31.2	-	-
Lac^{15}	169.9	67.6	16.4	-	-	-	-	-	-	-
Mle^{26}	170.6	55.2	36.7	24.8	23.1, 21.6	-	-	31.2	-	-
Phm^{37}	169.6	70.9	36.9	126.7	130.4	115.6	150.3	-	49.4	66.9
Mle^{48}	171.0	54.1	37.1	24.6	21.8, 23.4	-	-	30.5	-	-

Table S 4. Assignment of the major (asymmetric) and minor (symmetric) conformation of emodepside (**2**) in CD_3OH referenced to the residual CH_3OH shift in the solvent set to 3.33 ppm. * indicates non-assignable signals due to overlap and n.d. indicates signals that could not be detected. Mor1 is the C/H next to the oxygen in the morpholine ring, and Mor2 is the C/H next to the nitrogen in the morpholine ring.

	Hα	Hβ	Hγ	Hδ	Hε	HMe	HMor1	HMor2
Lac^{1}	5.18	0.94	-	-	-	-	-	-
Mle^{2}	4.78	1.88, 1.76	1.58	1.06, 1.00	-	2.84	-	-
Phm^{3}	5.79	3.11, 3.02	-	7.19	6.92	-	3.12-3.14*	3.83
Mle^{4}	5.40	1.65	0.85	0.84	-	2.87	-	-
Lac^{5}	5.55	1.40	-	-	-	-	-	-
Mle^{6}	5.45	1.75	1.35	0.96, 0.91	-	2.94	-	-
Phm^{7}	5.71	3.11, 3.03	-	7.19	6.92	-	3.12-3.14*	3.83
Mle^{8}	5.23	1.57, 1.50	0.92	0.83, 0.80	-	3.00	-	-
Lac^{15}	5.36	1.37	-	-	-	-	-	-
Mle^{26}	5.33	1.65	1.11	0.89, 0.87	-	2.97	-	-
Phm^{37}	5.68	3.01	-	7.18	6.92	-	3.12	3.83
Mle^{48}	5.11	1.65	1.40	0.92, 0.89	-	2.92	-	-

	C	Cα	Cβ	Cγ	Cδ	Cε	*Cζ*	CMe	CMor1	CMor2
Lac^{1}	173.1	68.5	15.8	-	-	-	-	-	-	-
Mle^{2}	170.9	57.2	37.6	24.7	20.2, 22.3	-	-	28.6	-	-
Phm^{3}	171.8	71.2	36.5	125.5	130.0	115.5	150.6-150.7*	-	49.1-49.2*	66.6
Mle^{4}	169.4	54.2	36.5	24.0	22.5, 20.1	-	-	30.0	-	-
Lac^{5}	172.1	67.1	16.1	-	-	-	-	-	-	-
Mle^{6}	169.6	54.1	37.2	24.8	22.5, 19.7	-	-	29.7	-	-
Phm^{7}	171.8	70.9	36.7	125.8	130.0	115.5	150.6-150.7*	-	49.1-49.2*	66.6
Mle^{8}	170.7	54.0	36.0	23.6	22.4, 20.3	-	-	30.6	-	-
Lac^{15}	171.9	68.3	15.4	-	-	-	-	-	-	-
Mle^{26}	n.d.	54.2	36.8	24.3	22.3, 20.4	-	-	31.3	-	-
Phm^{37}	170.8	71.3	36.4	126.4	130.0	115.6	150.5	-	49.3	66.6
Mle^{48}	170.4	n.d.	n.d.	24.7	22.4, 20.1	-	-	30.3	-	-

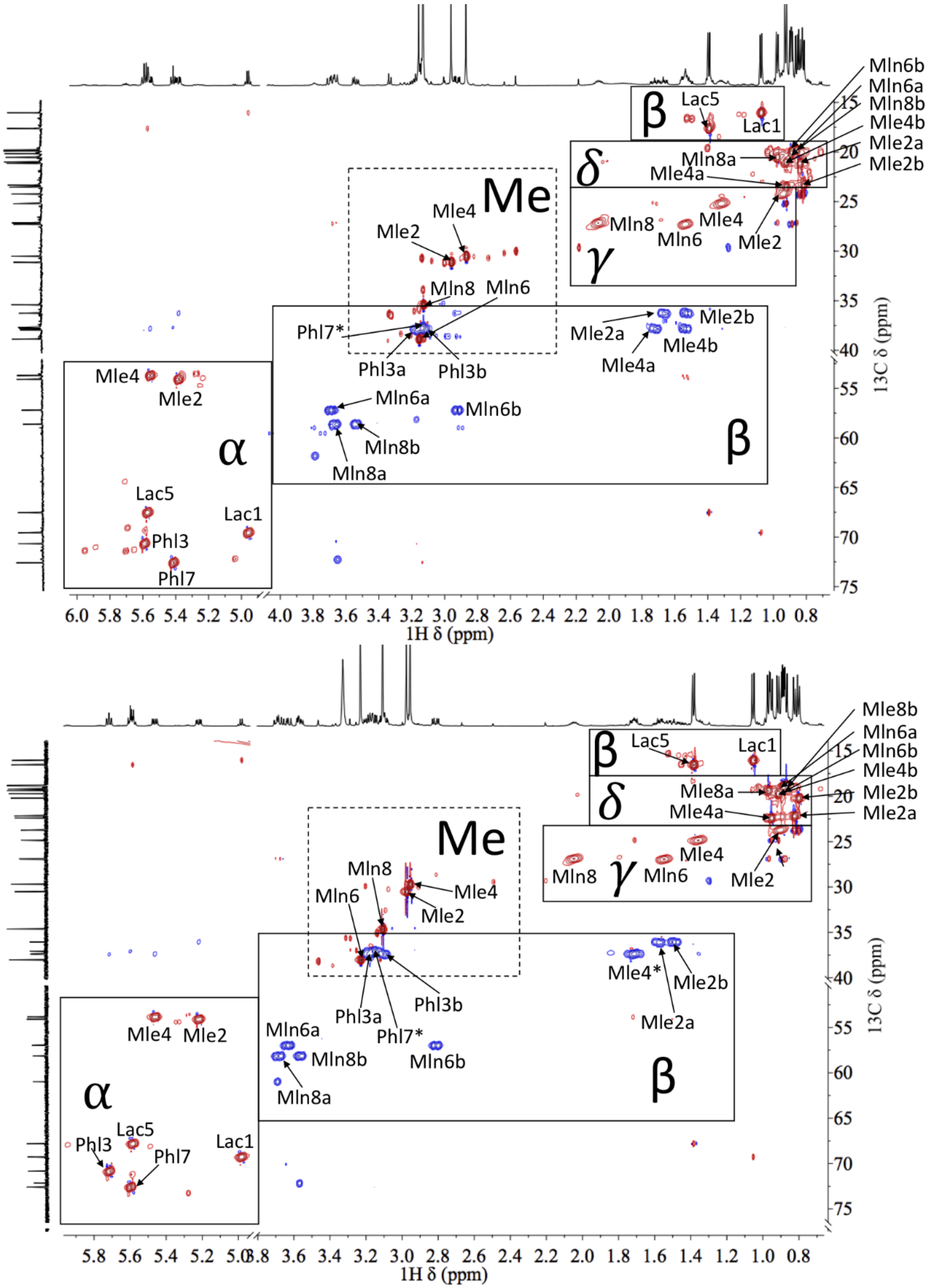

Figure S 3. ^{13}C-HSQC spectra of bis-aza-PF1022A analog (**8**) in $CDCl_3$ (top) and in CD_3OH (bottom). Empty regions are cut out for clarity.

Table S 5. Assignment of the major conformation (asymmetric) as well as assignment of Hα chemical shifts for the minor conformation (symmetric) denoted with ‘ of the bis-aza PF1022A analog (**8**) in $CDCl_3$ referenced to the residual $CHCl_3$ shift in the solvent set to 7.28 ppm.

		Hα	Hβ	Hγ	Hδ	Hε	Hζ	HMe
Lac^{1}		4.96	1.08	-	-	-	-	-
Mln^{2}		-	3.67, 3.35	2.07	0.98, 0.86	-	-	3.13
Phl^{3}		5.42	3.14	-	7.25	7.34	7.26	-
Mln^{4}		-	3.69, 2.93	1.54	0.89, 0.89	-	-	3.16
Lac^{5}		5.57	1.39	-	-	-	-	-
Mle^{6}		5.56	1.72, 1.54	1.32	0.92	-	-	2.87
Phl^{7}		5.59	3.18, 3.13	-	7.26	7.31	7.29	-
Mle^{8}		5.38	1.67, 1.54	0.94	0.83, 0.82	-	-	2.96
Phl$^{3'}$		5.81						
Lac$^{5'}$		5.28						
Mle6’		3.68						
Phl7’		5.99						
Mle8’		5.01						
	C	Cα	Cβ	Cγ	Cδ	Cε	*Cζ*	CMe
Lac^{1}	173.4	69.6	16.0	-	-	-	-	-
Mln^{2}	154.7	-	58.6	27.2	20.5, 19.8	-	-	35.4
Phl^{3}	168.7	72.6	37.7	134.9	129.4	128.7	127.1	-
Mln^{4}	154.0	-	57.2	27.3	20.2, 19.8	-	-	38.9
Lac^{5}	171.0	67.6	17.7	-	-	-	-	-
Mle^{6}	170.2	53.7	37.8	25.2	23.6, 21.0	-	-	30.5
Phl^{7}	170.2	70.7	38.0	135.4	129.5	128.5	127.4	-
Mle^{8}	171.5	54.1	36.3	24.3	21.2, 23.3	-	-	31.1

Table S 6. Assignment of the major conformation of the bis-aza PF1022A analog (**8**) in CD_3OH referenced to the residual CH_3OH shift in the solvent set to 3.33 ppm.

		Hα	Hβ	Hγ	Hδ	Hε	Hζ	HMe
Lac[1]		4.98	1.06	-	-	-	-	-
Mln[2]		-	3.69, 3.57	2.05	0.97, 0.87	-	-	3.11
Phl[3]		5.60	3.16	-	7.34	7.34	7.29	-
Mln[4]		-	3.64, 2.98	1.55	0.89, 0.88	-	-	3.23
Lac[5]		5.59	1.38	-	-	-	-	-
Mle[6]		5.46	1.72	1.36	0.95, 0.92	-	-	2.96
Phl[7]		5.72	3.19, 3.10	-	7.32	7.32	7.28	-
Mle[8]		5.22	1.58, 1.49	0.90	0.82, 0.81	-	-	2.98
	C	Cα	Cβ	Cγ	Cδ	Cε	*Cζ*	CMe
Lac[1]	174.2	69.3	16.1	-	-	-	-	-
Mln[2]	154.7	-	58.2	26.9	19.4, 18.8	-	-	34.7
Phl[3]	169.7	72.6	37.1	134.8	129.3	128.4	127.1	-
Mln[4]	153.9	-	57.0	27.0	18.9, 19.2	-	-	38.1
Lac[5]	172.3	67.9	16.6	-	-	-	-	-
Mle[6]	169.9	53.9	37.4	24.9	22.4, 19.8	-	-	29.7
Phl[7]	171.9	70.9	37.5	135.1	129.4	128.3	127.0	-
Mle[8]	171.2	54.1	36.1	23.8	22.2, 20.3	-	-	30.5

Table S 7. ^{15}N chemical shifts of **1**, **2** and **8** in $CDCl_3$ and CD_3OH. n.d. indicates signals that could not be detected.

	Mle^2 N	Mle^2 Nα	Mle^4 N	Mle^4 Nα	Mle^6 N	Mle^8 N	Mle^{26} N	Mle^{48} N	Mor N
PF1022A $CDCl_3$	107.4	-	110.6	-	104.8	112.6	110.2	111.4	-
PF1022A CD_3OH	110.5	-	114.2	-	107.2	115.6	n.d.	n.d.	-
Emodepside $CDCl_3$	107.4	-	110.7	-	104.9	112.7	110.3	111.3	103.1
Emodepside CD_3OH	110.6	-	114.6	-	107.3	115.9	n.d.	n.d.	103.0
bis-aza PF1022A analogue $CDCl_3$	95.2	122.5	94.5	123.9	104.1	112.7	n.d.	n.d.	-
bis-aza PF1022A analogue CD_3OH	96.3	122.5	96.8	123.6	106.5	115.2	n.d.	n.d.	-

Identification of Additional Conformers Based on Exchange, Exemplified for Compound 1

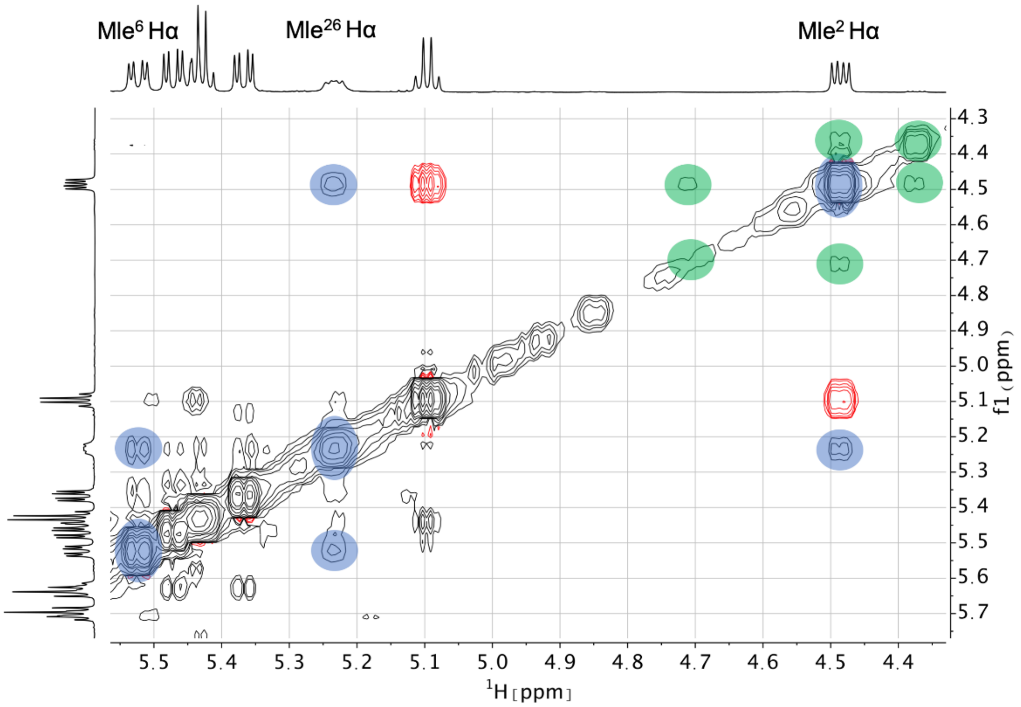

Figure S 4. Detail of ROESY spectrum showing the exchange between Mle^{2} Hα and Mle^{26} Hα as well as Mle^{6} Hα and Mle^{26} Hα of PF1022A (**1**) in chloroform (blue). ROE-peaks (red) have opposite phase relative to the diagonal whereas EXSY peaks have the same phase (black). Based on additional exchange peaks to Mle^{2} Hα, at least two additional conformers with very low intensity could be identified.

Plots for Fitting T_2 Relaxation Times of PF1022A (1) in Chloroform

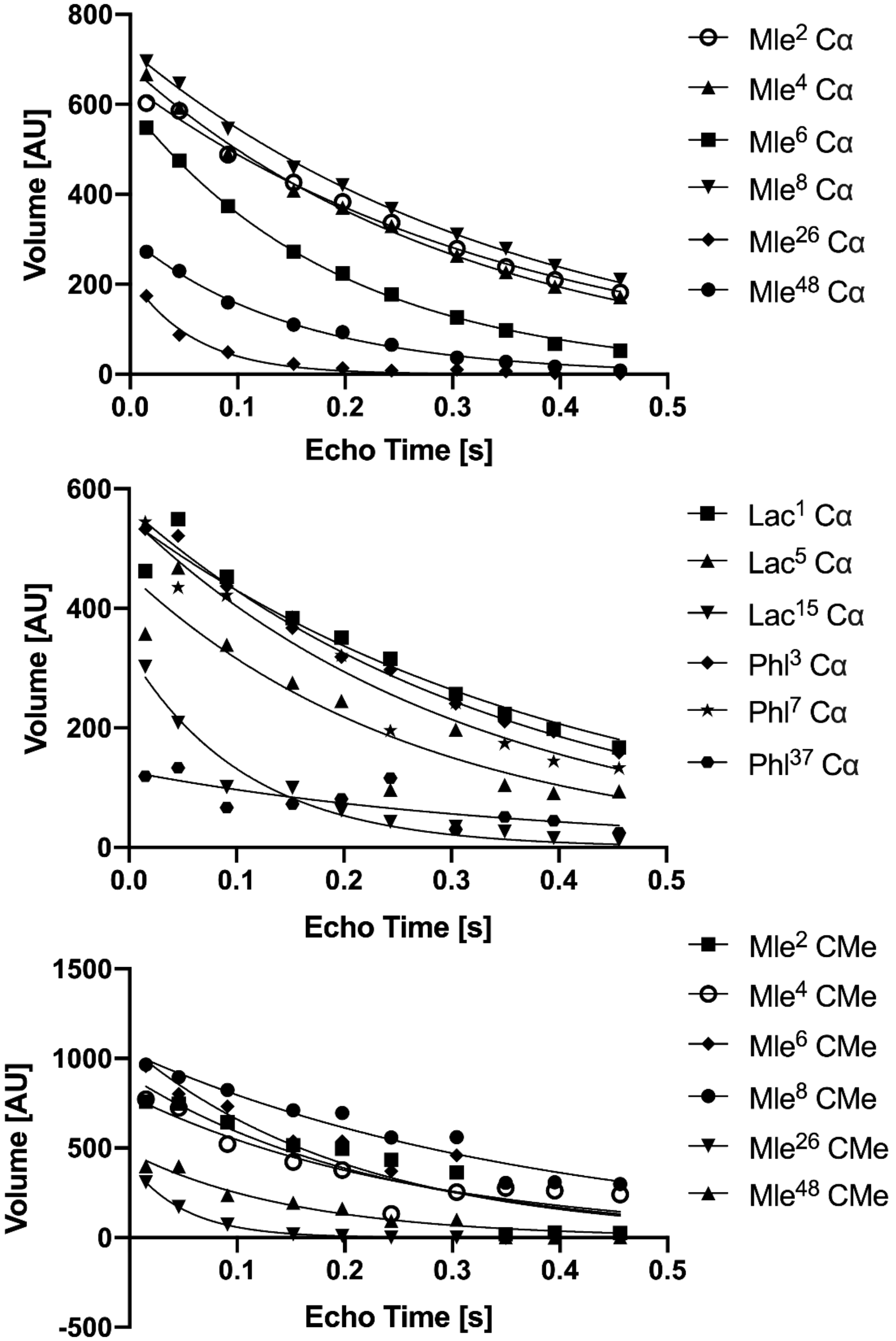

Figure S 5. Volumes extracted from the CPMG ^{13}C HSQC spectra plotted against the echo time to obtain the corresponding T_2 relaxation times for PF1022A (**1**) in chloroform. The plots were created with Prism.

Extracted Volumes and M_0 Values Used for Calculation of Site-to-Site Exchange Rates for compounds 1 (A), 2 (B), 8 (C) and 11 (D) in chloroform:

$$A = \begin{pmatrix} \frac{77.9}{1} & \frac{1.52}{1.5} & \frac{1.55}{1.5} \\ \frac{1.59}{1} & \frac{236}{1.5} & \frac{0}{1.5} \\ \frac{1.75}{1} & \frac{0}{1.5} & \frac{200}{1.5} \end{pmatrix} \qquad B = \begin{pmatrix} \frac{65.8}{1} & \frac{0.93}{1.8} & \frac{1.18}{1.8} \\ \frac{1.00}{1} & \frac{232}{1.8} & \frac{0}{1.8} \\ \frac{1.32}{1} & \frac{0}{1.8} & \frac{216}{1.8} \end{pmatrix}$$

$$C = \begin{pmatrix} \frac{12.9}{1} & \frac{0.50}{9.8} \\ \frac{0.46}{1} & \frac{387}{9.8} \end{pmatrix} \qquad D = \begin{pmatrix} \frac{95}{1} & \frac{1.58}{1.5} & \frac{1.7}{1.5} \\ \frac{2.41}{1} & \frac{235}{1.5} & \frac{0}{1.5} \\ \frac{1.82}{1} & \frac{0}{1.5} & \frac{218}{1.5} \end{pmatrix}$$

MSM Building

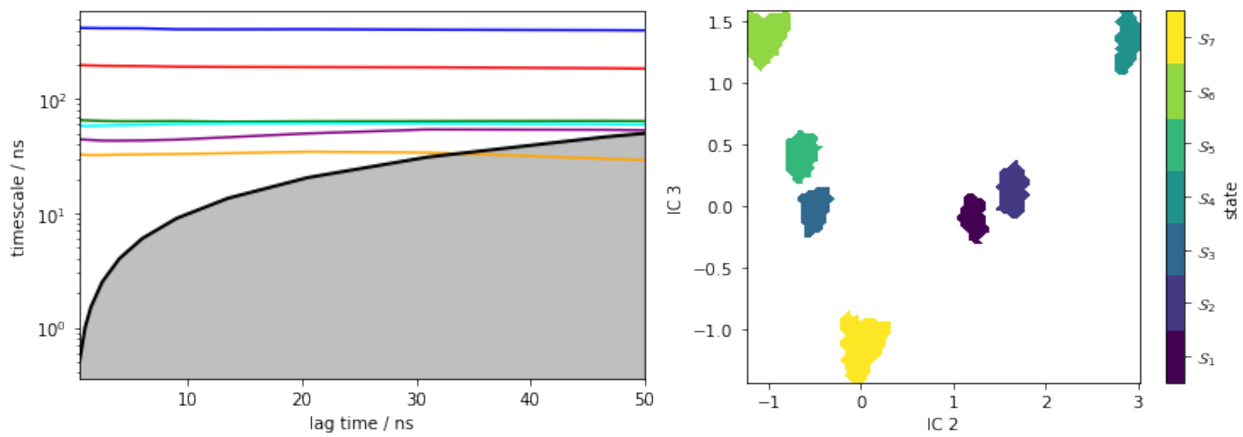

Figure S 6. (Left): Implied timescales for the MSM of the symmetric subset of PF1022A (**1**) in chloroform. Six slow transitions were observed. (Right): The seven conformational states of the symmetric subset plotted with the second and third TICA element. The first TICA element describes mainly the dihedral undergoing *cis-trans* isomerization and is thus not relevant for the symmetric subset.

Table S 8. Stationary distribution and corresponding free energies of the seven conformational states in the MSM of the symmetric subset of PF1022A (**1**) in chloroform.

State	Stationary distribution [%]	Estimated free energy [kT]
1	3.1	3.5
2	6.5	2.7
3	7.4	2.6
4	11.3	2.2
5	11.9	2.1
6	16.5	1.8
7	43.2	0.8

Titration of Emodepside with KSCN. General Procedure for the Fitting of Titration Data.

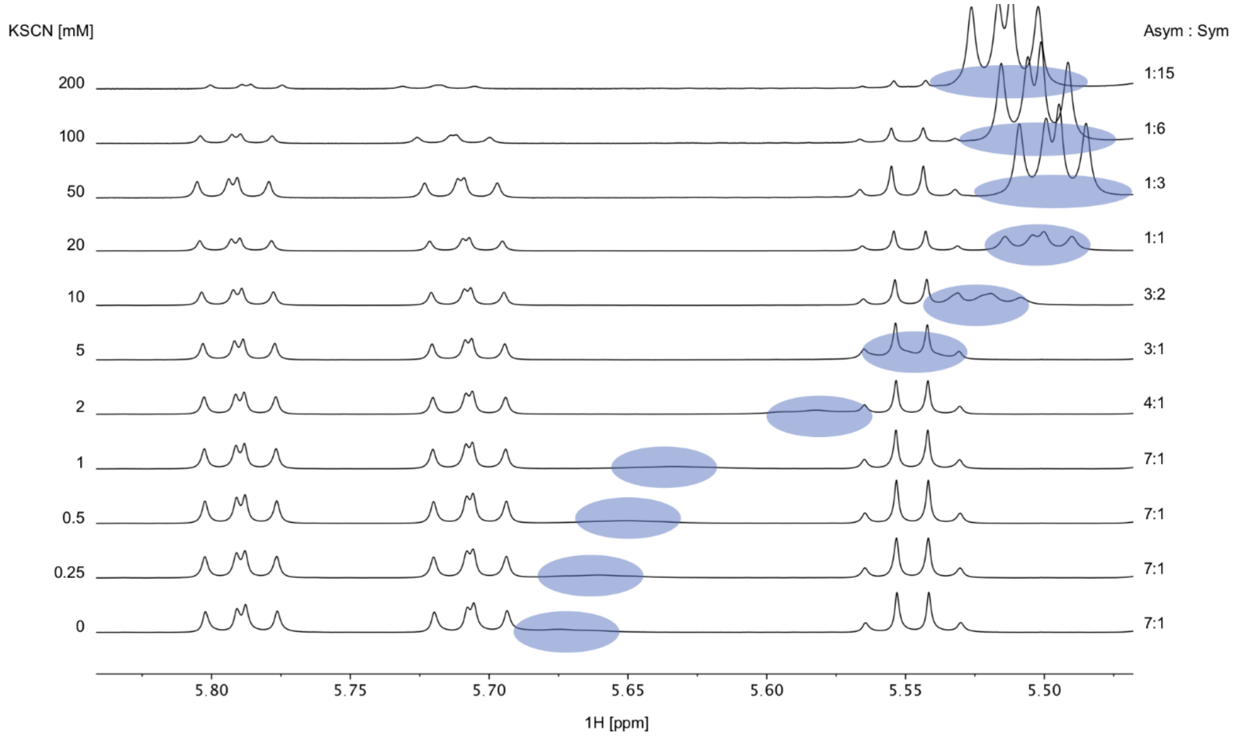

Figure S 7. Hα region of ^{1}H NMR spectra of 5 mM emodepside (**2**) in CD_3OH at different KSCN concentrations. Chemical shift changes were observed for the symmetric conformation, best seen for the signal of the Hα proton in residue Phm[37] (blue labels). In addition, a change in the ratio between the symmetric and asymmetric conformation is observed. Also the asymmetric conformation shows small chemical shift changes at high salt concentrations.

The concentration of the symmetric conformation in dependence of the salt concentration was fitted with the following equation (damped logistic growth function):

$$[P_{free}] = [P_{all}] - ([P_{all}] - [P_0]) * e^{\frac{-a*[S]}{[S]+b}}$$

where $[P_{free}]$ is the concentration of the free symmetric peptide, $[P_{all}]$ the total peptide concentration, $[P_0]$ the symmetric peptide concentration without salt, $[S]$ the salt concentration, and a and b are the fitting parameters.

The chemical shift change in dependence of the salt concentration was fitted using the following equations assuming a fast equilibrium between the free symmetric species, the 1:1 complex and a 2:1 complex:

$$\delta_{obs} = \delta_P - \frac{(\delta - \delta_{PS}) * [S_{tot}] * K_1 * [P_{free}] + 2 * (\delta_P - \delta_{P2S}) * [S_{tot}] * K_1 * K_2 * [P_{free}]^2}{P_0 * \left(1 + K_1 * [P_{free}] + * K_1 * K_2 * [P_{free}]^2\right)}$$

$$0 = K_1 * K_2 * [P_{free}]^3 + K_1 * (2 * K_2 * [S_{tot}] - K_2 * [P_{tot}] + 1) * [P_{free}]^2$$
$$+ (K_1 * ([S_{tot}] - [P_{tot}]) + 1) * [P_{free}] - [P_{tot}]$$

$$\text{with } K_1 = \frac{[PS]}{[P_{free}][S]} \text{ and } K_2 = \frac{[P_2S]}{[PS][S]}$$

where δ_{obs} corresponds to the observed chemical shift, δ_P is the chemical shift of the symmetric free peptide, δ_{PS} is the chemical shift of the 1:1 symmetric peptide-cation complex (*PS*), δ_{P2S} the shift of the 2:1 symmetric peptide-cation complex (P_2S), [P_{tot}] the total concentration of the peptide (all symmetric species), directly determined from the integral of the signal of the symmetric species in ^{1}H NMR spectrum, [S_{tot}] is the total salt concentration, [P_{free}] the free symmetric peptide concentration, and K_1 and K_2 are the equilibrium constants for the 1:1 and the 2:1 complexes. Fitting was done with R.

Experimental Results for mono-iodine analog (11):

Table S 9. Ratio between asymmetric and symmetric conformer in CD_3OH and $CDCl_3$ for compounds **11**.

Compound	Conformer ratio in CD_3OH (asymmetric : symmetric)	Conformer ratio in $CDCl_3$ (asymmetric : symmetric)
Mono-iodo analog (**11**)	5:1	3:1

Table S 10. Exchange rates between asymmetric and symmetric conformers of **11** in $CDCl_3$ determined from EASY-ROESY experiment with mixing time of 100 ms.

Compound	k_1 [s^{-1}]	k_2 [s^{-1}]	k_{ex} [s^{-1}]
Mono-iodo analog (**11**)	0.17	0.09	0.26

Table S 11. Change in ratio between asymmetric and symmetric conformer without salt and after addition of a 25-fold excess of CsSCN in CD_3OH.

Salt	Mono-iodo analog (**11**)
CsSCN	5:1 to 1:80

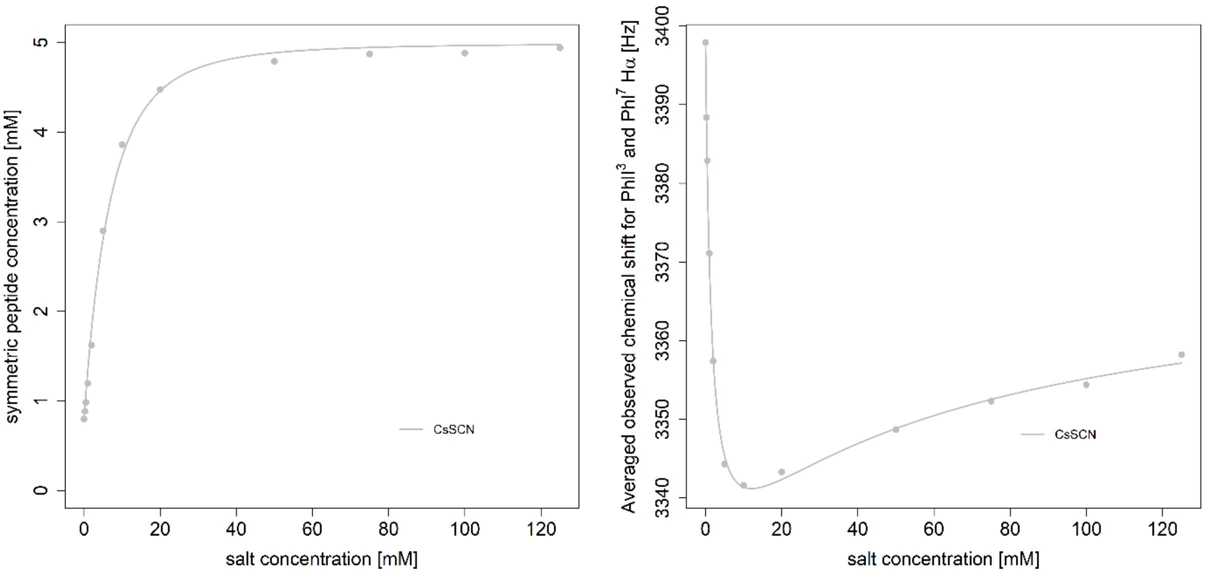

Figure S 8. Titration of 5 mM mono-iodine analog (**11**) with CsSCN in CD_3OH while the total volume was kept constant. (Left): Change in concentration of the symmetric conformation upon the addition of CsSCN. The data points were fitted with a damped logistic growth function. (Right): Change of the chemical shift of the averaged signal of $PhII^3$ and $PhII^7$ Hα proton of the symmetric conformation as a function of the salt concentration.

Permeability Data PAMPA for compounds 1, 2 and 8

Table S 12. PAMPA results of compounds **1**, **2** and **8** with and without the addition of 100 mM KCl. Carbamazepine was used as control for the PAMPA and showed permeabilities between 7.94 and 11.1 * 10^{-6} cm s^{-1} and recovery rates between 85.4 and 199.9 %.

Compound	Average Permeability with standard deviation in brackets [10^{-6} cm s^{-1}]	Average Recovery Rate [%]
PF1022A (**1**)	0.06 [0.03]	18.4
Emodepside (**2**)	0.00 [0.00]	78.7
Bis-aza analog (**8**)	0.08 [0.03]	26.2
PF1022A (**1**) + 100 mM KCl	0.02 [0.01]	57.4
Emodepside (**2**) + 100 mM KCl	0.00 [0.00]	27.3
Bis-aza analog (**8**) + 100 mM KCl	0.00 [0.00]	6.8
